# Natural cyclopeptide RA-V inhibits the NF-κB signaling pathway by targeting TAK1

**DOI:** 10.1038/s41419-018-0743-2

**Published:** 2018-06-18

**Authors:** Zhe Wang, Simeng Zhao, Lihua Song, Yuzhi Pu, Qiang Wang, Guangzhi Zeng, Xing Liu, Ming Bai, Shao Li, Fabao Gao, Lijuan Chen, Chen Wang, Ninghua Tan

**Affiliations:** 1 School of Traditional Chinese Pharmacy & State Key Laboratory of Natural Medicines, China Pharmaceutical University, Nanjing, 211198 China; 2 State Key Laboratory of Phytochemistry and Plant Resources in West China, Kunming Institute of Botany, Chinese Academy of Sciences, Kunming, 650201 China; 3 State Key Laboratory of Biotherapy/Collaborative Innovation Center of Biotherapy and Cancer Center, West China Hospital of Sichuan University, Chengdu, 610041 China; 4 State Key Laboratory of Cell Biology, Shanghai Institute of Biochemistry and Cell Biology, Chinese Academy of Sciences, Shanghai, 200031 China; 5 MOE Key Laboratory of Bioinformatics and Bioinformatics Division, TNLIST/Department of Automation, Tsinghua University, Beijing, 100084 China

## Abstract

Rubiaceae-type cyclopeptides (RAs) are a type of plant cyclopeptides from the *Rubia* that have garnered significant attention owing to their unique bicyclic structures and amazing antitumour activities. Our recent work has shown that RAs suppress inflammation and angiogenesis and induce apoptosis. However, the underlying mechanism and targets remained unknown. Nuclear factor κB (NF-κB) signaling pathway plays a critical role in these biological processes, prompting us to investigate whether and how RAs affect this pathway. By screening compound libraries using NF-κB-dependent luciferase reporter, we observed that RA-V is the best NF-κB inhibitor. Further experiments demonstrated that RA-V interrupted the TAK1–TAB2 interaction and targeted TAK1 in this pathway. Moreover, RA-V prevented endotoxin shock and inhibited NF-κB activation and tumor growth in vivo. These findings clarify the mechanism of RA-V on NF-κB pathway and might account for the majority of known bioactivities of RA-V, which will help RA-V develop as new antiinflammatory and antitumour therapies.

## Introduction

**Fig. 1:**
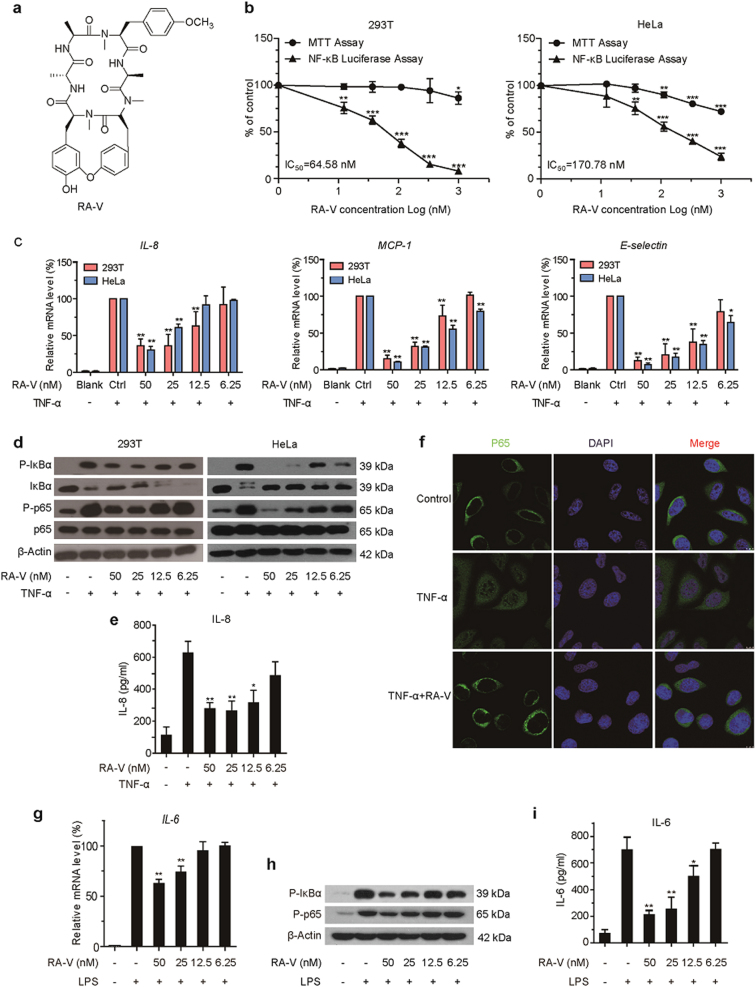
RA-V inhibits TNF-α- and LPS-induced activation of NF-κB. **a** Chemical structure of RA-V. **b** RA-V inhibited the NF-κB signaling pathway in a dose-dependent manner. HEK293T or HeLa cells were transfected with the 5 × κB-luciferase and pTK-Renilla reporters. Twenty-four hours after transfection, the cells were incubated with various concentrations of RA-V for 6 h, and then treated with 10 ng/mL TNF-α for 2 h before the luciferase activity assay and MTT assay. **c** RA-V reduced TNF-α-induced expression of NF-κB target genes. HEK293T or HeLa cells were treated with various concentrations of RA-V for 24 h and stimulated with 10 ng/mL TNF-α for 2 h. The expression of the NF-κB target genes, *IL-8*, *MCP-1,* and *E-selectin* was measured by quantitative RT-PCR and normalized to *GADPH* expression. **d** RA-V inhibited TNF-α-induced p65 phosphorylation, IκBα phosphorylation and IκBα degradation. HEK293T or HeLa cells were incubated with various concentrations of RA-V for 24 h and treated with 10 ng/mL TNF-α for 10 min. The cell lysates were prepared and subjected to western blot analysis with the indicated antibodies. **e** RA-V inhibited TNF-α-induced IL-8 production. HeLa cells were treated with various concentrations of RA-V for 12 h before treatment with 10 ng/mL TNF-α for 2 h. The culture supernatant was collected and subjected to ELISA analysis. **f** RA-V inhibited the TNF-α-induced nuclear translocation of p65. HeLa cells were incubated with 200 nM RA-V for 6 h, treated with 10 ng/mL TNF-α for 15 min, and then subjected to immunocytochemical analysis. **g** RA-V reduced LPS-induced *IL-6* expression. RAW264.7 cells were treated with various concentrations of RA-V for 24 h and stimulated with 1 μg/mL LPS for 3 h. The expression of *IL-6* was measured. **h** RA-V inhibited LPS-induced p65 phosphorylation, IκBα phosphorylation. RAW264.7 cells were incubated with various concentrations of RA-V for 24 h and treated with 1 μg/mL LPS for 3 h. The protein expression was measured. **i** RA-V inhibited LPS-induced IL-6 production. RAW264.7 cells were treated with various concentrations of RA-V for 12 h before treatment with 1 μg/mL LPS for 3 h. The expression of IL-6 was measured. The data in **b**, **c**, **e**, **g** and **i** are presented as the means ± S.D. from three independent experiments. *, *p* *<* 0.05; **, *p* *<* 0.01; ***, *p* *<* 0.001

Natural products derived from medicinal herbs have been a rich source of leading compounds and have played a vital role in drug discovery for centuries^1,2^. The roots and rhizomes of the *Rubia* plants, including *Rubia cordifolia*, were recorded as a traditional Chinese medicine in Chinese Pharmacopeia and have been widely used for the treatment of menoxenia, rheumatism, contusions, and tuberculosis. Rubiaceae-type cyclopeptides (RAs), quinones, and triterpenes have been isolated from the *Rubia* plants^3–6^. Among them, RA-VII, a Rubiaceae-type cyclopeptide, was reported to have undergone phase I clinical trials at the NCI as an anticancer drug in Japan in 1990s^3^. To date, 56 RAs have been isolated from higher plants^6–10^, and these compounds have attracted considerable attention over the past three decades because of their unique bicyclic structures and amazing antitumour activities in vitro and in vivo, particularly RA-VII and RA-V (Fig. 1a).

Nuclear factor κB (NF-κB), a family of important transcription factors, was discovered as a protein that binds the immunoglobulin κ light chain gene enhancers in B cells^11^. The NF-κB family contains five proteins in mammals, including RelA (p65), RelB, c-Rel, NF-κB1, and NF-κB2, which are sequestered in the cytoplasm as hetero- or homodimers. There are two distinct pathways: canonical and non-canonical pathways^12^. In the canonical NF-κB pathway the inhibitory protein IκBα is phosphorylated, ubiquitinated and degraded, which leads to the nuclear translocation of the NF-κB complex and regulates the expression of the target genes^12^. Mounting evidence has indicated that the NF-κB signaling pathway plays a critical role in many biological processes and controls the expression of over 500 target genes that are involved in cell proliferation, apoptosis, angiogenesis, metastasis, and inflammation^13,14^. Abnormal activation of NF-κB pathway is frequently happened in many diseases, such as cancer, arthritis, and diabetes^15,16^. Thus, NF-κB has been an important therapeutic target, particularly in cancer, which has prompted significant effort to identify moderators. At present, > 700 inhibitors of the NF-κB signaling pathway have been reported, but only a few have been developed into drugs^14,17^. For example, Bortezomib (Velcade), a reversible 26 S proteasome inhibitor, exerts its inhibitory activity on NF-κB at nanomolar concentrations and has been approved by the US FDA for the treatment of multiple myeloma^18^.

Transforming growth factor beta-activated kinase 1 (TAK1/MAP3K7), an evolutionarily conserved member of the mitogen-activated protein kinase kinase kinase family, is a key kinase upstream of NF-κB which can be activated by various stimuli and plays an essential role in this pathway^19^. Accumulating studies have suggested an association between dysregulated expression of TAK1 and many human diseases, including cancer^20^. Therefore, TAK1 is becoming an attractive therapeutic target for cancer treatment.

To identify potential natural NF-κB inhibitors, we screened several small molecule libraries of ~ 200 compounds primarily derived from plants. Of the inhibitors that we identified, RA-V showed the best inhibitory effect on NF-κB pathway. Our previous studies have demonstrated that RA-V has antiinflammatory activity by inhibiting NO production and tumor necrosis factor (TNF)-α-induced NF-κB activation^21;^ RA-V significantly suppresses angiogenesis by downregulating ERK1/2 phosphorylation in HUVEC and HMEC-1 endothelial cells^22;^ RA-V kills human breast cancer cells by inducing mitochondria-mediated apoptosis and inhibits cell adhesion and invasion via the PI3K/AKT and NF-κB signaling pathways^23,24.^ Notably, the potential roles for RAs in cancer therapy have been highlighted^25^. However, the underlying mechanisms and specific targets in the NF-κB pathway remained unknown. We proposed that RA-V might exert its effects by regulating NF-κB pathway. Therefore, we investigated how RA-V inhibits NF-κB activation in the current study and identified that TAK1 is the RA-V target in this pathway. Moreover, RA-V powerfully blocks endotoxin shock and represses NF-κB activation and tumor growth in vivo. These discoveries not only clarify the mechanism of RA-V in NF-κB pathway, but also might account for the majority of known bioactivities of RA-V, which will contribute to the future development of RA-V as new therapeutic agents for the treatment of cancer and inflammatory diseases.

## Results

### RA-V inhibits TNF-α- and LPS-induced activation of NF-κB

To discover small molecules that inhibit the NF-κB signaling pathway, we performed a cell-based screen using small molecule libraries, including a library of RAs consisting of 29 natural and 5 synthetic RAs. We found that RAs were one of potential natural NF-κB inhibitors, particularly RA-V (Fig. 1a and Supplementary Figure 1), and observed that RA-V dose-dependently inhibited TNF-α-induced activation of an NF-κB-dependent luciferase reporter in HEK293T and HeLa cells. In contrast, we observed only a slight antiproliferative effect from RA-V at high concentrations (Fig. 1b and Supplementary Figure 2). To further confirm the inhibitory activity of RA-V on the NF-κB pathway, we investigated the effect of RA-V on NF-κB target genes by quantitative RT-PCR. As shown in Fig. 1c, RA-V dose-dependently decreased the TNF-α-induced expression of *IL-8*, *MCP-1*, and *E-selectin*. Moreover, we examined the effect of RA-V on the expression of NF-κB-associated proteins. As expected, western blot analysis demonstrated that RA-V also had dose-dependent inhibitory effects on TNF-α-induced IκBα phosphorylation, IκBα degradation, and p65 phosphorylation (Fig. 1d). In addition, we found that RA-V inhibited the TNF-α-induced production of IL-8 in a dose-dependent manner using enzyme-linked immunosorbent assay (ELISA) (Fig. 1e). The key step in canonical NF-κB activation is the translocation of p65/p50 dimer from the cytoplasm to the nucleus^12^, prompting us to perform an immunofluorescence assay. As showed in Fig. 1f, RA-V significantly blocked TNF-α-induced p65 nuclear translocation. Besides, RA-V also inhibited lipopolysaccharide (LPS)-induced interleukin (IL)-6 production, IκBα phosphorylation and p65 phosphorylation in a dose-dependent manner in RAW264.7 cells (Fig. 1g–i). Collectively, these results demonstrated that RA-V is a potent inhibitor of the NF-κB signaling pathway, with IC_50_ values of 64.58 nM in HEK293T cells and 170.78 nM in HeLa cells, and that RA-V probably acts upstream of p65 nuclear translocation.

### RA-V prevents endotoxin shock and inhibits NF-κB activation in vivo

The above results prompted us to explore the in vivo effect of RA-V on the NF-κB signaling pathway. We extended our study to the mouse endotoxin shock model, in which mice were intraperitoneally injected with LPS (10 mg/kg or 20 mg/kg) and evaluated for changes by examining physiological parameters induced by the inflammatory response^26^.

**Fig. 2:**
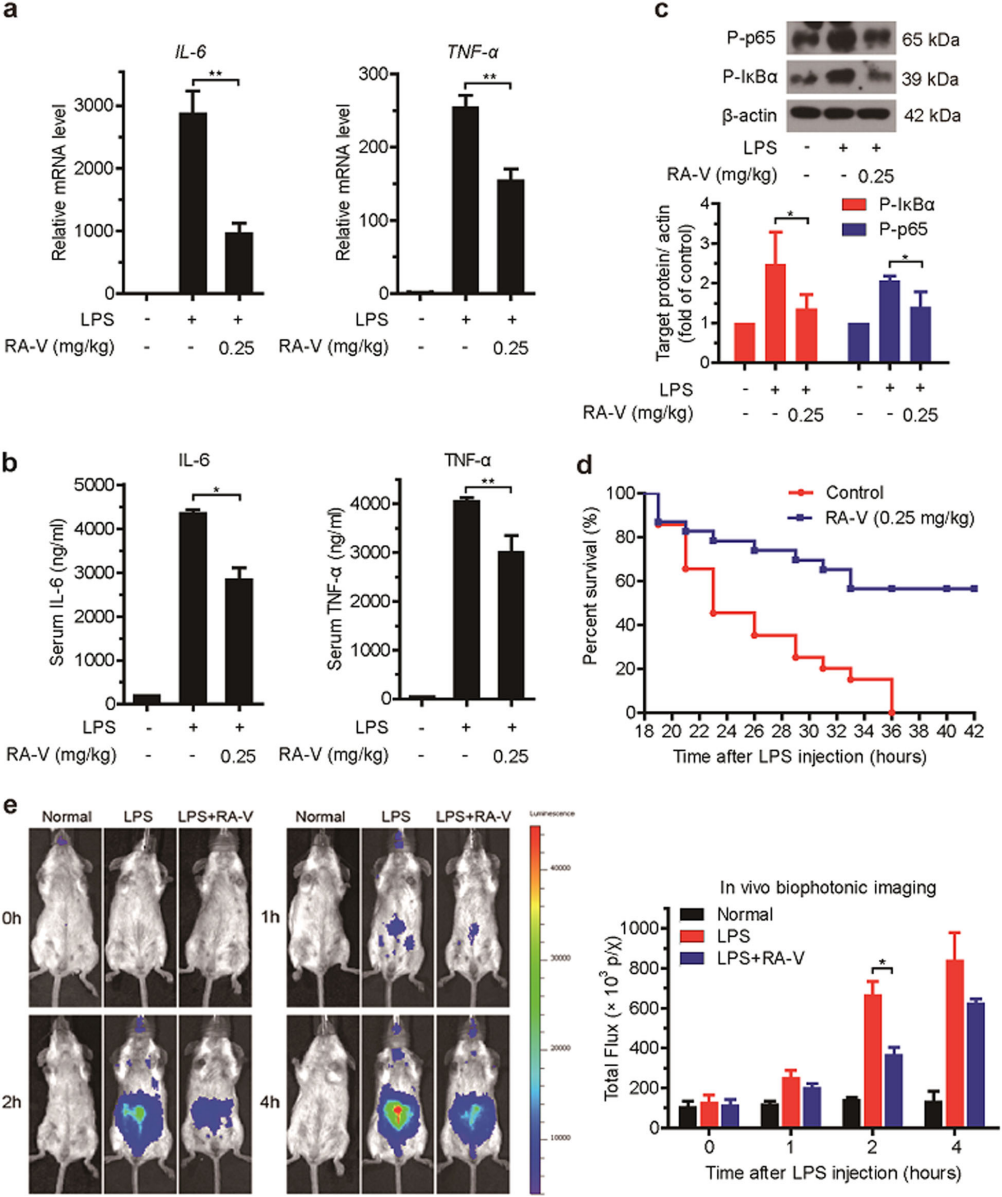
RA-V prevents endotoxic shock and inhibits NF-κB activation *in vivo*. **a**–**c** RA-V prevents endotoxic shock in vivo. Mice (*n* = 6 per group) were intravenously injected with RA-V micelles (0.25 mg/kg body weight) or control micelles 52, 28, and 4 h before an intraperitoneal injection of LPS (10 mg/kg). One hour later, the relative levels of *IL-6* and *TNF-α* mRNAs in the liver were assessed by quantitative RT-PCR (**a**), the serum IL-6 and TNF-α levels were quantified by ELISA (**b**), and the expression of p65 and IκBα phosphorylation in the liver were measured by immunoblot analysis **c**. **d** RA-V improved animal survival. Mice (*n* = 20 per group) were intravenously injected with RA-V micelles (0.25 mg/kg body weight) or control micelles 52, 28, and 4 h before intraperitoneal injection of LPS (20 mg/kg). Animal survival was recorded in 2–3 hours intervals. **e** RA-V inhibits NF-κB activation in vivo. The NF-κB-luc transgenic mice (n = 4 per group) were intravenously injected with RA-V micelles (0.25 mg/kg body weight) or control micelles 52, 28, and 4 h before intraperitoneal injection with LPS (10 mg/kg). NF-κB-luc transgenic mice were photographed at different time points and representative images are shown. The bioluminescent signals from all of the mice were detected and quantified. The data are presented as the means ± S.D. *, *p* *<* 0.05; **, *p* *<* 0.01

First, the mice were intravenously injected with RA-V micelles (0.25 mg/kg body weight) or control micelles at three time points: 52, 28, and 4 h before they were intraperitoneally injected with phosphate-buffered saline (PBS) or LPS (10 mg/kg). One hour after LPS injection, we examined proinflammatory cytokines (IL-6 and TNF-α) production at the transcriptional and translational levels. The expression of *IL-6* and *TNF-α* mRNAs was significantly decreased in liver tissue of the RA-V-treated mice (Fig. 2a). The LPS- and RA-V-treated mice consistently exhibited reduced expression of IL-6 and TNF-α in the serum (Fig. 2b). Further analysis demonstrated that the phosphorylation of IκBα and p65 was clearly inhibited in the liver tissue of RA-V-treated mice (Fig. 2c). In addition, RA-V also significantly improved the survival of mice treated with a lethal dose of LPS (20 mg/kg). As expected, after receiving the LPS injection, all of the control mice died within 36 h, whereas 60% of the RA-V-treated mice survived (Fig. 2d). To further confirm the in vivo effect of RA-V on the NF-κB pathway, we performed the endotoxin shock model on NF-κB-luciferase transgenic mice. The in vivo bioluminescence imaging showed that RA-V inhibited NF-κB activation, particularly 2 h after LPS injection (Fig. 2e). Collectively, these results demonstrated that RA-V prevents endotoxic shock in mice and inhibits activation of the NF-κB signaling pathway in vivo.

### RA-V represses NF-κB activation upstream of the IKK protein complex

**Fig. 3:**
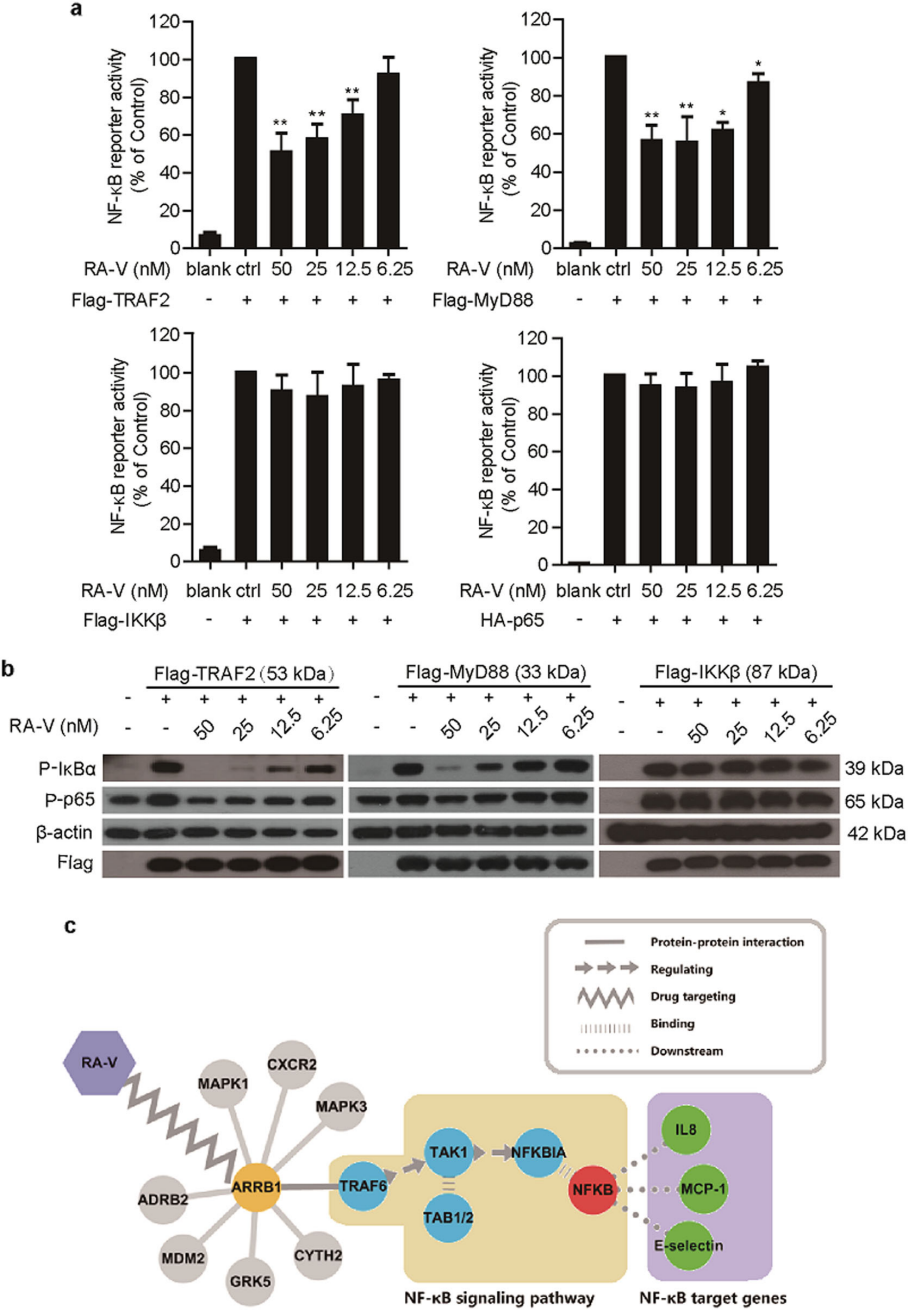
RA-V represses NF-κB activation upstream of the IKK protein complex. **a** RA-V inhibited the TRAF2- and MyD88-induced expression of the NF-κB reporter in a dose-dependent manner. The indicated plasmids were transfected into HEK293T cells together with the 5 × κB-luciferase and pTK-Renilla reporters. Twenty-four hours after transfection, the cells were incubated with various concentrations of RA-V for 48 h before luciferase assays were performed. **b** RA-V inhibited TRAF2- and MyD88-induced p65 phosphorylation and IκBα phosphorylation in a dose-dependent manner. HEK293T cells were transfected with TRAF2, MyD88, IKKβ, or p65 for 24 h and then treated with various concentrations of RA-V for 48 h. The cell lysates were immunoblotted with the indicated antibodies. **c** RA-V likely exerts its inhibitory activity on the NF-κB pathway by acting on the TRAF6-TAK1–TAB1/2 complex. Using drugCIPHER, ARRB1 is a potential target of RA-V, as ARRB1 ranks 44th of 13388 genome-wide candidates. TRAF6-TAK1–TAB1/2 complex is a key link that connects ARRB1 and the NF-κB signaling pathway by searching the STRING database. The data in **a** and **b** are presented as the means ± S.D. from three independent experiments. *, *p* *<* 0.05; **, *p* *<* 0.01

TNF-α and LPS have been shown to activate the canonical NF-κB pathway through sequential interactions with TRAF2/MyD88, TAK1, IKK and p65^15^. To determine where RA-V acts in this pathway, TRAF2, MyD88, IKKβ, or p65 plasmid was transiently transfected into HEK293T cells together with the 5 × κB-lucieferase and pTK-Renilla reporters. RA-V dose-dependently suppressed expression of the NF-κB-regulated reporter in cells transfected with the TRAF2 or MyD88 plasmid, but had no effect on expression in cells transfected with IKKβ or p65 (Fig. 3a). Western blots were performed to investigate the effect of RA-V on NF-κB-associated proteins in HEK293T cells transfected with the TRAF2, MyD88, or IKKβ plasmid. As shown in Fig. 3b, RA-V dose-dependently inhibited p65 phosphorylation and IκBα phosphorylation in cells overexpressing TRAF2 or MyD88, but had no effect on expression in cells transfected with the IKKβ plasmid. These findings indicated that RA-V might act upstream of the IKK protein complex. Moreover, we used a computational method to predict where RA-V is likely to act in this pathway. Using drugCIPHER^27^, a state-of-the-art network pharmacology tool that infers target profiles for drugs and small molecules in a genome-wide scale, and searching the STRING database^28^, we found that RA-V likely exerted its inhibitory activity on NF-κB signaling pathway by acting on TRAF6-TAK1–TAB1/2 complex (Fig. 3c). Taken together, these results confirmed that RA-V exerts its inhibitory activity on the NF-κB signaling pathway upstream of the IKK protein complex.

### RA-V blocks the interaction between TAK1 and TAB2

**Fig. 4:**
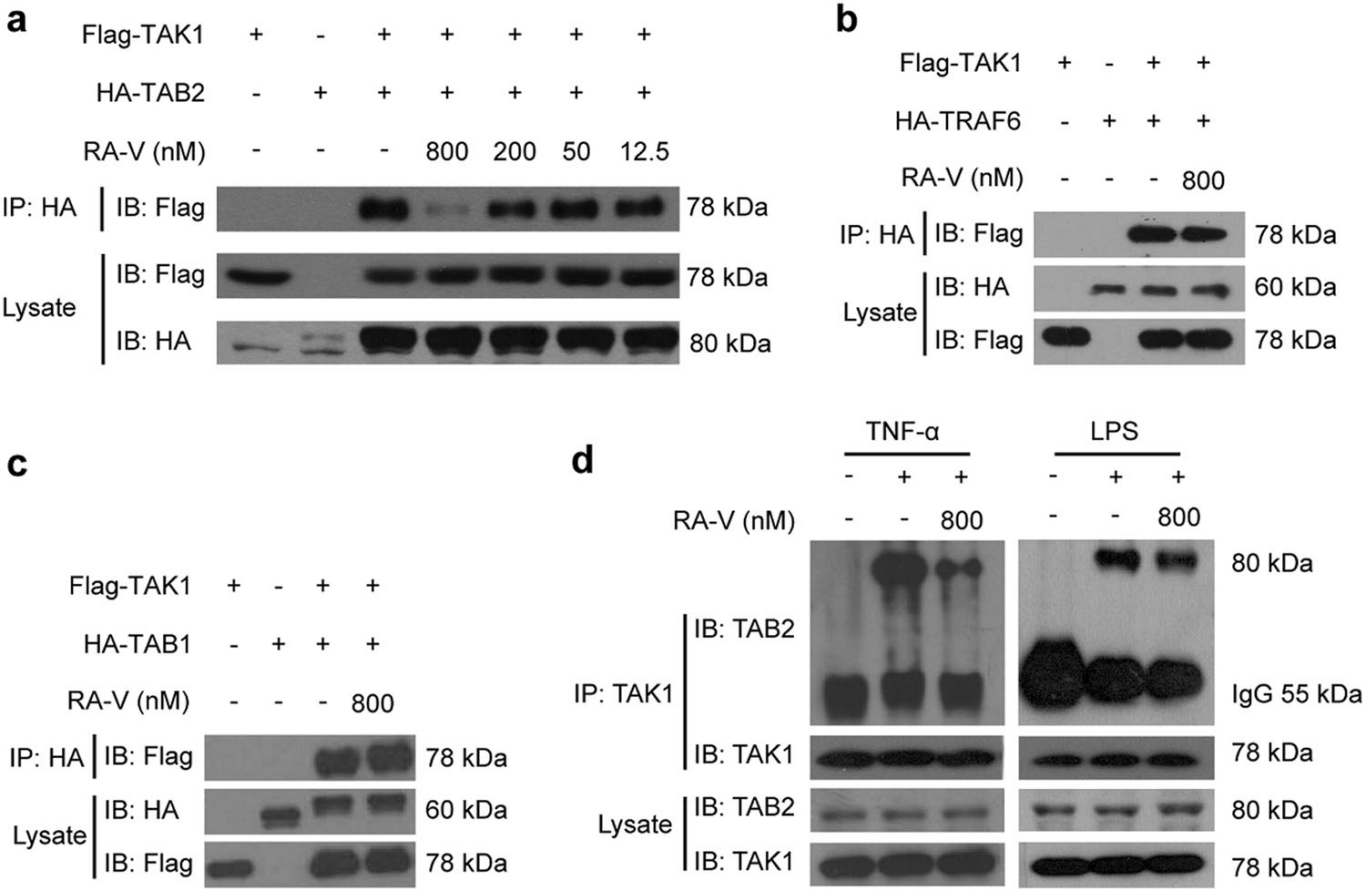
RA-V blocks the interaction between TAK1 and TAB2. **a** RA-V blocked the exogenous TAK1–TAB2 interaction. HEK293T cells were transfected with Flag-TAK1 and HA-TAB2 for 24 h and then incubated with various concentrations of RA-V for 6 h. The cell lysates were immunoprecipitated with HA antibody and then immunoblotted with the indicated antibodies. **b**, **c** RA-V does not disrupt TAK1–TAB1 or TAK1–TRAF6 interactions. HEK293T cells were transfected with Flag-TAK1 and HA-TRAF6 or HA-TAB1 for 24 h and then incubated with RA-V (800 nM) for 6 h. The cell lysates were immunoprecipitated with HA antibody, and immunoblots were performed with the indicated antibodies. **d** RA-V blocked the endogenous TAK1–TAB2 interaction. HEK293T (left) or RAW264.7 (right) cells were incubated with 800 nM RA-V for 6 h and stimulated with 10 ng/mL TNF-α for 2 h or 1 μg/mL LPS for 3 h, respectively. The cell lysates were immunoprecipitated with TAK1 antibody or control IgG and then immunoblotted with the indicated antibodies. Each experiment was repeated at least three times

TAK1, a serine/threonine kinase, is a member of the MAP3K family that regulates several signaling pathways, including the NF-κB signaling pathway^11^. In many different cell types, TAK1-binding protein 1 (TAB1) and TAB2 form a stable complex with TAK1 in response to various stimuli and play a key role in the activation of NF-κB^29,30^. TNF receptor-associated factor 6 (TRAF6), an E3-ligase, is positioned upstream of TAK1 and recruits it in response to various stimuli that induce NF-κB activation^31^. To explore the mechanism how RA-V inhibits NF-κB activation upstream of the IKK protein complex, we analyzed the effects of RA-V on TAK1–TAB1, TAK1–TAB2, and TAK1–TRAF6 interactions using co-immunoprecipitation. We found that RA-V dose-dependently blocked the interaction between exogenously expressed TAK1 and TAB2 (Fig. 4a), but not between TAK1–TAB1 or TAK1–TRAF6 (Fig. 4b, c). To determine whether RA-V perturbed the interaction between endogenous TAK1 and TAB2, we performed additional co-immunoprecipitation experiments to examine the effect of RA-V on the TNF-α- or LPS-induced interaction between TAK1 and TAB2. As shown in Fig. 4d, RA-V inhibited the interaction between TAK1 and TAB2. Collectively, these results revealed that RA-V blocks the interaction between TAK1 and TAB2.

### RA-V targets the TAK1 protein

**Fig. 5:**
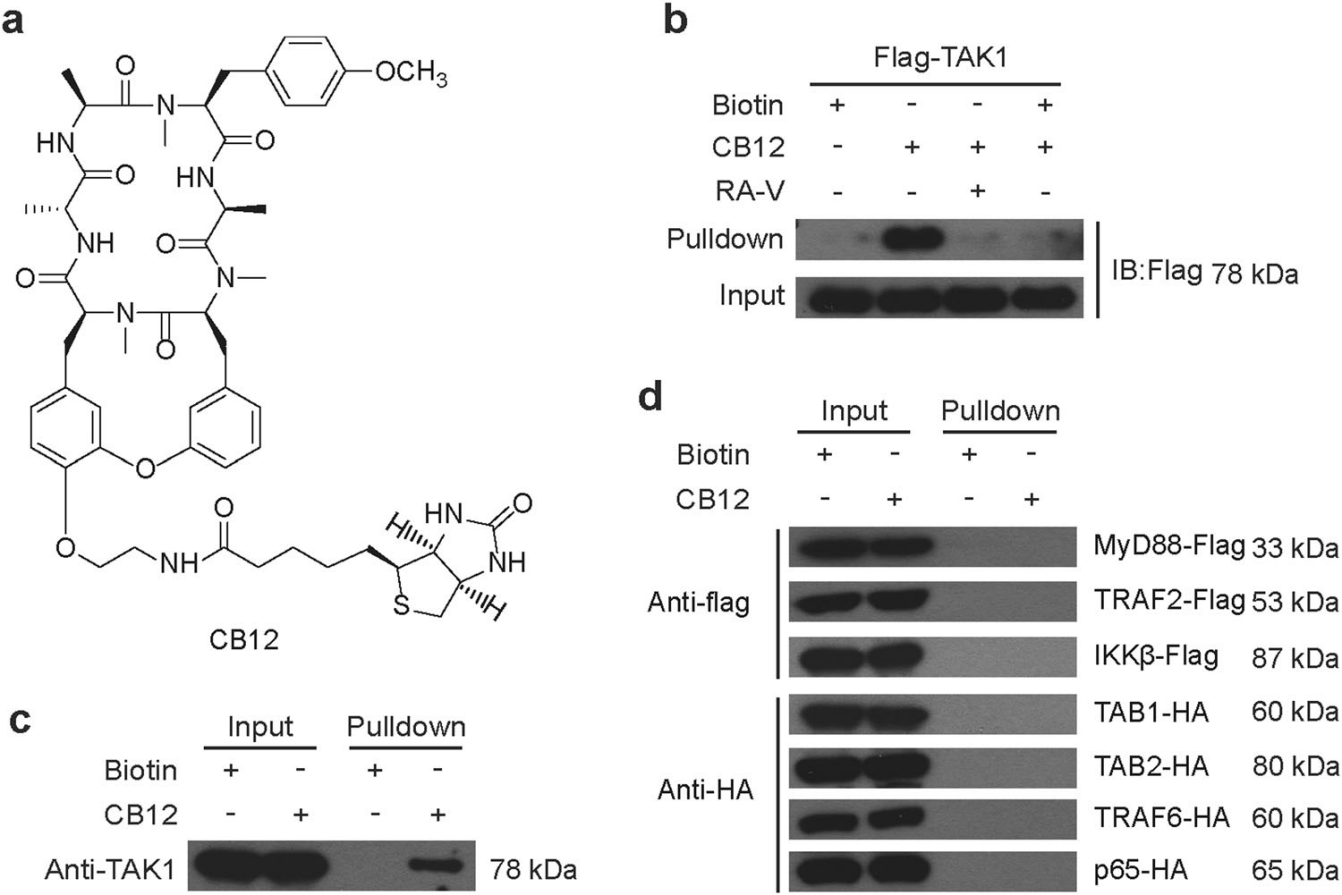
RA-V targets the TAK1 protein. **a** Chemical structure of CB12. **b** CB12 binds the exogenous TAK1. HEK293T cells were transfected with TAK1 for 24 h, then the cell lysates were incubated with biotin or CB12 followed by streptavidin agarose pull-down. The immunoprecipitates were incubated with a 10-fold excess of RA-V or biotin and western blotted with Flag antibody. **c** CB12 binds the endogenous TAK1. HEK293T cell lysates were incubated with CB12 or biotin and precipitated with streptavidin agarose, followed by western blotting with TAK1 antibody. **d** CB12 did not bind other key proteins of the NF-κB signaling pathway. HEK293T cells were transfected with the indicated plasmids for 24 h. The cell lysates were pulled down with streptavidin agarose and then immunoblotted with HA or Flag antibody. Each experiment was repeated at least three times

We attempted to synthesize chemical probes to identify the potential RA-V targets responsible for its inhibitory effect on NF-κB activation. Synthetic derivatives were designed based on the structure-activity relationship between RAs and NF-κB (Supplementary Figure 1) and the modifiable sites of the RA-V structure. We selected the 2-alanine and 6-tyrosine amino acids as the modification sites, and a series of analogs (CB1-12, Supplementary Figure 3a) were synthesized, whose NF-κB inhibitory activities were evaluated using the NF-κB-dependent luciferase reporter. We found that NF-κB inhibition was variably decreased for CB1-12, particularly CB1 through CB5, which exhibited IC_50_ values > 20 µM. Fortunately, CB12 (Fig. 5a and Supplementary Figure 3b), a biotin-tagged RA-V, retained the ability to inhibit NF-κB signaling pathway with an IC_50_ value of 2.91 μM (Supplementary Figure 3c–d). Therefore, we chose CB12 as a chemical probe to identify potential targets of RA-V.

Because RA-V blocks the interaction between TAK1 and TAB2, we explored whether TAK1 or TAB2 could bind to CB12. HEK293T cells were transfected with Flag-TAK1 or HA-TAB2 for 24 h. The cell lysates were incubated with free biotin or CB12, and the mixtures were precipitated with streptavidin agarose followed by western blot analysis. We found that Flag-TAK1, not HA-TAB2 was effectively pulled down by CB12 and the band could be competed away by higher concentrations of unlabeled RA-V (Fig. 5b), indicating that TAK1 likely binds to RA-V. To further investigate whether RA-V binds to endogenous TAK1, we also performed a pull-down assay to confirm the interaction between CB12 and TAK1. As shown in Fig. 5c, the binding between CB12 and endogenous TAK1 was presented. Besides, no interaction was found between CB12 and the other major NF-κB-related proteins, including MyD88, TRAF2, TRAF6, TAB1, TAB2, IKKβ, and p65 (Fig. 5d). These results demonstrated that TAK1 is the cellular RA-V target in the NF-κB signaling pathway.

### RA-V binds the kinase domain of TAK1

**Fig. 6:**
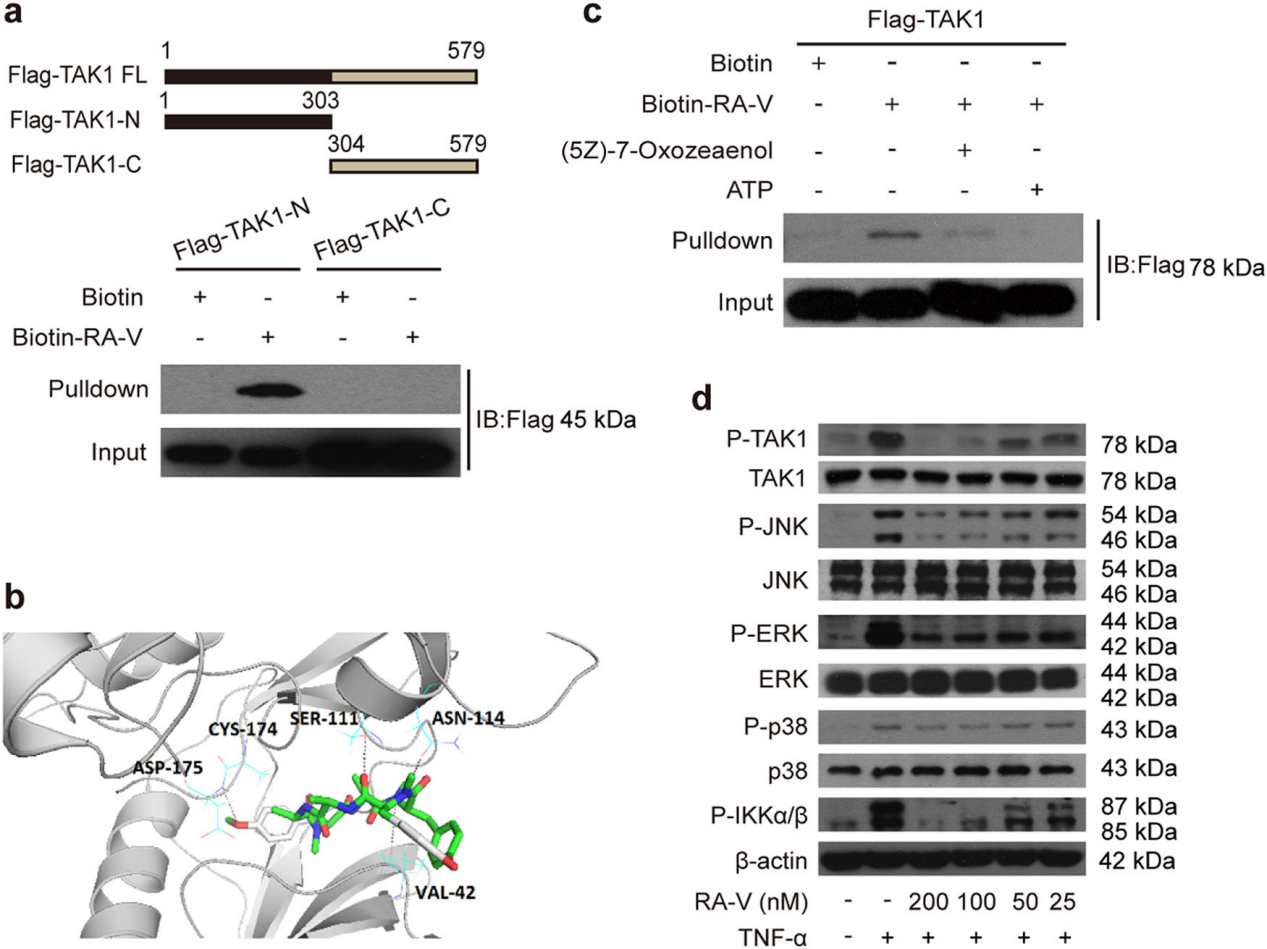
RA-V binds the kinase domain of TAK1. **a** CB12 directly binds to the TAK1 kinase domain. HEK293T cells were transfected with TAK1-N or TAK1-C for 24 h. The cells lysates were incubated with CB12 or biotin and precipitated with streptavidin-coated sepharose and were analyzed by immunoblotting with Flag antibody. **b** The model for RA-V binding to the crystal structure of the TAK1–TAB1 fusion protein (PDB 4GS6). The blue dotted lines represent the hydrogen bonds between RA-V (green) and TAK1–TAB1. **c** RA-V probably binds the ATP-binding pocket of TAK1. HEK293T cells were transfected with TAK1 for 24 h, and then the cell lysates were incubated with biotin or CB12 before precipitation with streptavidin agarose. The immunoprecipitates were treated with ATP or (5Z)-7-oxozeaenol and western blotted with Flag antibody. **d** RA-V inhibits TAK1, JNK, ERK, p38, and IKKα/β phosphorylation. HeLa cells were incubated with various concentrations of RA-V for 12 h and then treated with 10 ng/mL TNF-α for 10 min. The cell lysates were prepared and western blotted with the indicated antibodies. Each experiment was repeated at least three times

To explore the mechanism in detail, we generated two Flag-tagged TAK1 truncation mutants, one of which encoded the kinase domain. We observed that RA-V only bound the kinase domain of TAK1 (Fig. 6a). Next, we modeled the RA-V-TAK1 complex. The Auto Dock vina program was used to predict the binding site for RA-V on the TAK1 protein (TAK1–TAB1 complex, PDB: 4GS6). A detailed analysis of the generated models showed that RA-V might bind to the ATP-binding pocket of TAK1 (affinity free energy: 9.8 kcal/mol), and hydrogen bond interactions between RA-V and Asp-175, Asn-114, Ser-111, Val-42 were observed (Fig. 6b). An additional pharmacophore screen using the pharmacophore generated by the RA-V-TAK1 complex showed that the model gathered RA molecules with NF-κB inhibitory activities, indicating the accuracy of the predicted binding cavity (Supplementary Figure 4). Furthermore, we found that (5Z)-7-Oxozeaenol, a potent ATP-competitive irreversible inhibitor of TAK1^32,33^, effectively competed with the interaction between CB12 and TAK1 (Fig. 6c) using a pull-down assay. ATP was used as a control. We also observed that RA-V inhibited TAK1 phosphorylation and the proteins that TAK1 directly or indirectly phosphorylates, including IKKα/β, JNK, ERK, and p38 (Fig. 6d). Collectively, these results suggested that RA-V binds the kinase domain of TAK1, and the binding sites probably located in the ATP-binding pocket.

### RA-V exerts antitumour effect by inhibiting NF-κB activation in vivo

NF-κB signaling pathway could promote cell survival by regulating target genes and always be activated aberrantly or constitutively in many tumor cells^14^. Comparing the results in Fig. 1 with Supplementary Figure 2, we found that the bioactivities against tumor cell proliferation and NF-κB activation of RA-V have good positive correlation in vitro, which drove us to investigate whether RA-V suppresses tumor growth in vivo by inhibiting NF-κB activation.

**Fig. 7:**
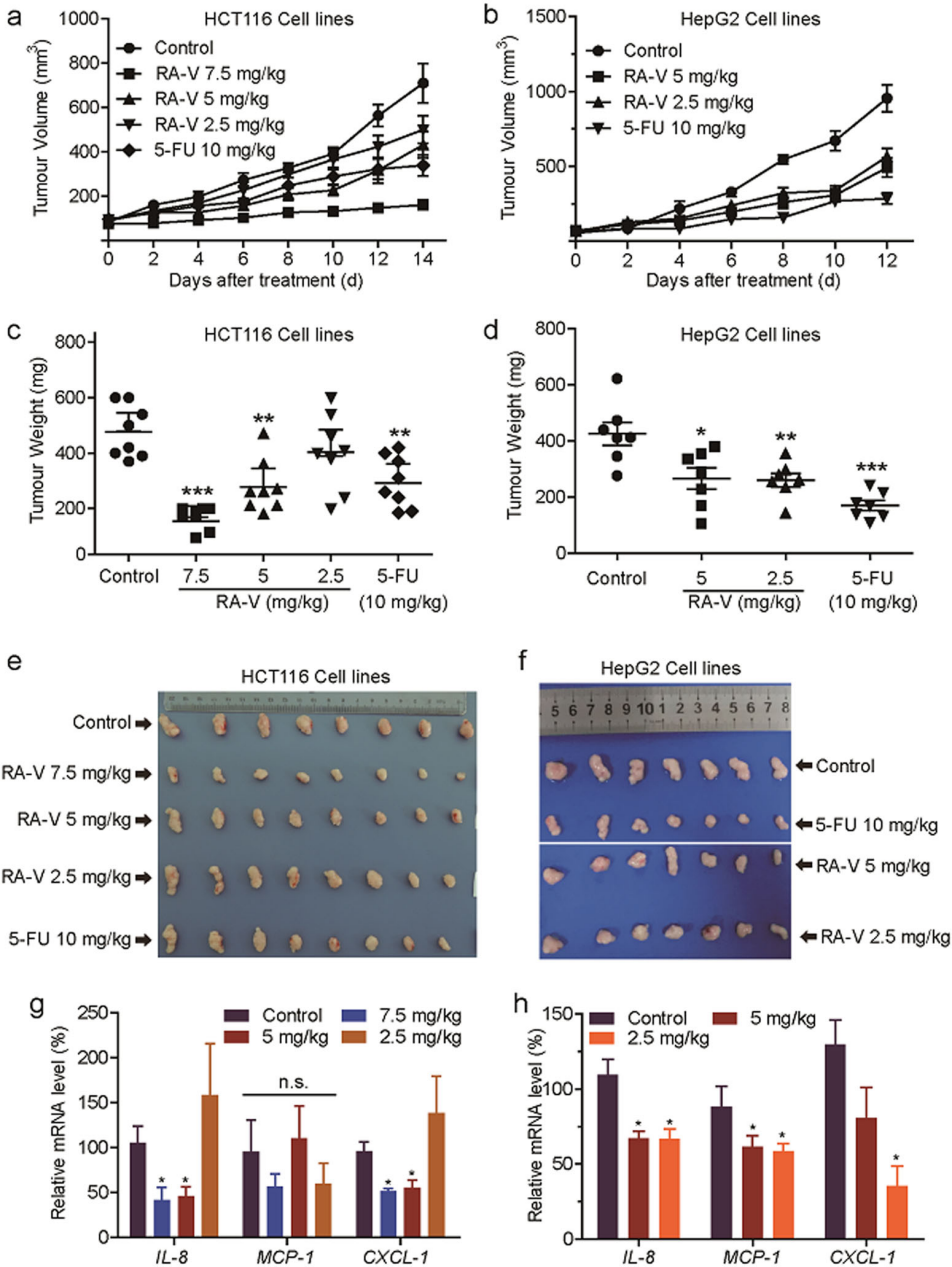
RA-V inhibits tumor growth and the expression of NF-κB target genes in a nude xenograft model. **a**–**d** Inhibition of the growth of HCT116 and HepG2 xenograft tumors by RA-V. Female athymic nude BALB/c mice bearing HCT116 (*n* = 8) or HepG2 (*n* = 7) xenograft tumors were intravenously injected with various concentrations of RA-V micelles or control micelles every other day. 5-FU (10 mg/kg) group as positive control. Effects of RA-V on the growth curves of subcutaneoue xenografts of HCT116 (**a**) and HepG2 (**b**) and effects on the tumor weight in the HCT116 (**c**) and HepG2 (**d**) models. **e**–**f** Tumors removed were photographed. **g**–**h** The expression of the NF-κB target genes, *IL-8*, *MCP-1,* and *CXCL-1*, in HCT116 (**g**) and HepG2 (**h**) tumor tissue from vehicle- and various concentrations of RA-V-treated group was determined by quantitative RT-PCR and normalized to *GADPH* expression. *, *p* *<* 0.05; **, *p* *<* 0.01; ***, *p* *<* 0.001

**Fig. 8:**
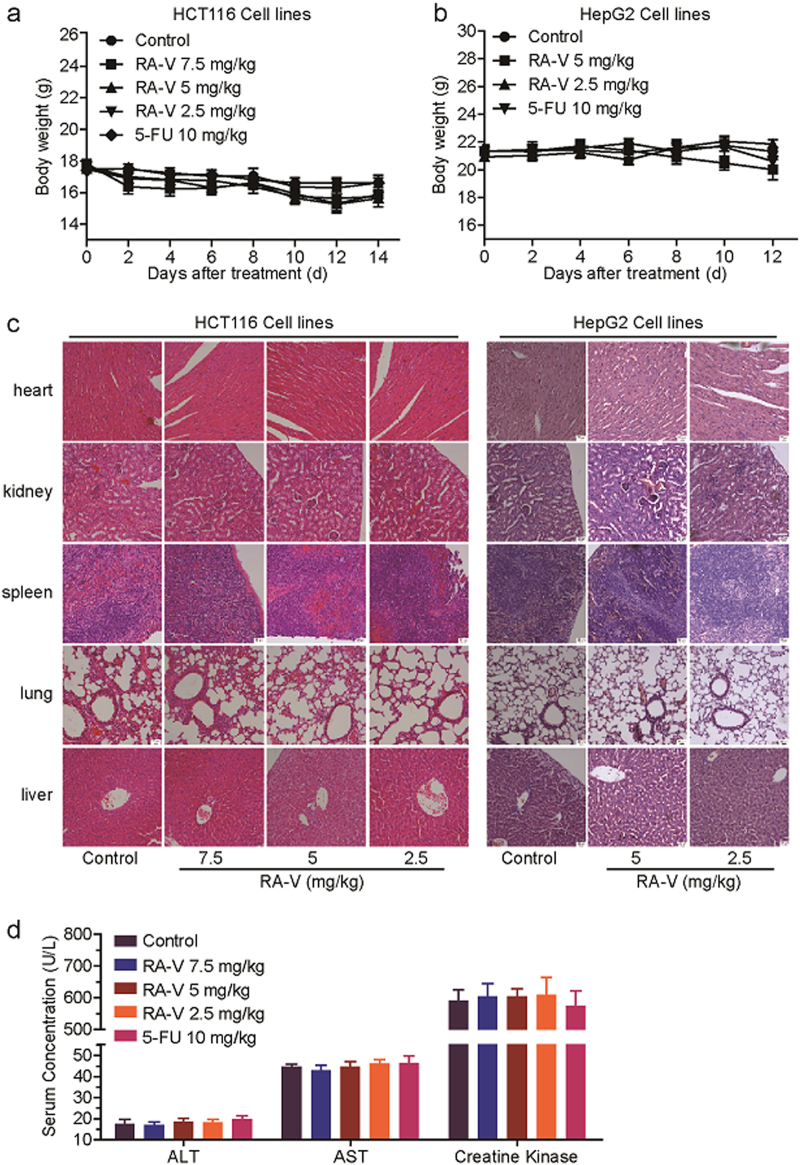
Analysis of potential side effects for treatment of RA-V. **a–b** The change curves of body weights of BALB/c bearing HCT116 (**a**) or HepG2 (**b**) xenograft tumors (*n* = 8 or 7). **c** Representative hematoxylin–eosin staining of heart, kidney, spleen, lung, and liver from vehicle- and various concentrations of RA-V-treated group. **d** The evaluation of serum ALT, AST, and creatine kinase for vehicle- and various concentrations of the RA-V-treated group

The antitumour effects in vivo were evaluated in HCT116 and HepG2 xenografts in nude mice. As shown in Fig. 7a, b, treatment with RA-V significantly reduced the growth rates of HCT116 and HepG2 tumor growth compared with the control by measuring tumor volume every other day. The tumor weight of mice treated with RA-V was also obviously lower than the control (Fig. 7c–f). To assess the possible toxicity of RA-V, body weight was measured every other day throughout the whole experiment. As shown in Fig. 8a, b, weight of mice in the RA-V-treated groups had no obvious changes in comparison with the control. We also investigated toxic pathological changes in main organs by hematoxylin–eosin staining. Microscopic examination demonstrated that no obvious toxic pathological changes were found in heart, kidney, spleen, lung and liver in the RA-V-treated groups compared with the control (Fig. 8c). The levels of serum ALT, AST and creatine kinase were also determined to assess the liver and heart toxicity, respectively. As expected, no significant changes were observed (Fig. 8d). Furthermore, quantitative RT-PCR analysis in tumor tissue from xenografts was performed to determine the mRNA levels of NF-κB target genes. As shown in Fig. 7g, h, the expression of *IL-8*, *MCP-1,* and *CXCL-1* were remarkable decreased in the RA-V-treated groups compared with the control. Taken together, these results suggested that RA-V exerts antitumour activity in vivo without any discernible side effects by inhibiting NF-κB activation.

## Discussion

According to the data from the World Health Organization (WHO), cancer, particularly of the lung, colon, and breast cancer, is one of leading causes of death in many countries and is still increasing^34^. To reduce the mortality rates, many studies have focused on the occurrence and development of cancer to identify key targets or signaling pathways as potential treatment options. Among them, the NF-κB signaling pathway has drawn considerable attention owing to its constitutive activation in many cancers, including both hematopoietic and solid malignancies^35^, prompting many researchers to develop NF-κB inhibitors. Although > 700 compounds have inhibitory effects on the NF-κB pathway, most of them block signaling downstream of IKK, and only a few have been included in clinical trials or used as clinical cancer remedies^14^. Therefore, the identification of novel NF-κB inhibitors with new targets is still needed.

To develop small molecules that inhibit the NF-κB signaling pathway, we first performed a cell-based screen using small molecule libraries (~ 200 compounds) and found that RAs exhibited powerful inhibitory activities on the NF-κB pathway. RAs that contain unique bicyclic structures are potential natural antitumour compounds. Among them, RA-VII was approved for phase I clinical trials by Japanese scientists in the 1990s, but its use was forcefully terminated owing to its toxicity and water solubility^3^. Nevertheless, much antitumour mechanistic investigations and new drug study of RAs have been performed during these years. Earlier studies indicated that RA-VII suppresses protein synthesis by binding to eukaryotic 80 S ribosomes^36^. Moreover, RA-V and RA-XII significantly inhibit NO overproduction and the inducible nitric oxide synthase^37^. Furthermore, RA-VII exhibits the ability to change the conformational structure of F-actin to induce G_2_ arrest and exhibits antiangiogenic activity in vitro and in vivo^38,39^. Our previous studies have also shown that RA-V has antiproliferative, antiinflammatory and anti-angiogenesis activities in many cell lines^21–25^. However, the underlying mechanisms and RA targets in the NF-κB pathway have remained unknown. Because the NF-κB signaling pathway is involved in many important biological processes, such as cell proliferation, apoptosis, and tumor metastasis, we investigated the inhibitory mechanisms and identified RA targets in the NF-κB pathway that might be responsible for most of the known activities of RAs reported to date, including the anti-proliferative, antiinflammatory, anti-angiogenesis, and apoptotic activities.

Then, we focused on RA-V, the best one among the 34 RA derivatives, with an IC_50_ value of 64.58 nM, as assessed by the NF-κB-dependent luciferase reporter in HEK293T cells. We confirmed that RA-V is a new natural inhibitor using subsequent in vitro and in vivo experiments and came to several conclusions: (a) Cells treated with various concentrations of RA-V exhibit decreased expression of TNF-α- and LPS-induced NF-κB-responsive genes. (b) RA-V inhibits the expression of TNF-α- and LPS-induced NF-κB-associated proteins in a dose-dependent manner. (c) The TNF-α-induced translocation of the p65/p50 heterodimer is blocked by RA-V. (d) In the LPS-induced endotoxic shock model, RA-V not only reduces the expression of proinflammatory cytokines and NF-κB-associated proteins but also consistently prolongs survival time. (e) The inhibitory activity of RA-V was also confirmed in NF-κB-luciferase transgenic mice by bioluminescence imaging in vivo. Thus, these results encouraged us to select RA-V as a representative compound for further analysis investigating inhibitory mechanisms and targets of the NF-κB signaling pathway.

Although it is believed that the activities of the IKK complex proteins and proteasome are important for NF-κB activation^40,41^, RA-V fails to inhibit their activities or expression. Therefore, we explored where RA-V acts in the NF-κB signaling pathway by biochemical and bioinformatics analyses. The results indicated that both TRAF2- and MyD88-induced NF-κB activation and the expression of NF-κB-associated proteins are prevented by RA-V, but not those induced by IKKβ and p65, indicating that RA-V acts upstream of IKK complex. The bioinformatics analysis also supports this interpretation. Therefore, RA-V exerts its inhibitory effect in a different way than most known NF-κB inhibitors.

TAK1, a member of the MAP3K family that is a kinase upstream of IKKβ in the NF-κB signaling pathway, has a critical role in the canonical pathway through its interaction with TAB1, TAB2, and TRAF6^29,30,42^. RA-V blocked both the endogenous and exogenous interaction between both TAK1 and TAB2. Based on the structure-activity relationship between RAs and NF-κB and the modifiable sites on the RA-V structure, we synthesized several different chemical probes to identify one (CB12) that was active. Using biotin-tagged RA-V (CB12) as a probe, which has an IC_50_ value of the 2.91 μM, we discovered that RA-V directly targets TAK1 and probably binds to its ATP-binding pocket, excluding the direct binding to other important NF-κB-associated proteins such as MyD88, TRAF2/6, and TAB1/2, which were also supported by computational study. In agreement with these results, RA-V inhibits the phosphorylation of proteins that are downstream of TAK1, such as IKKβ, JNK, ERK, and p38^43,44^. Unfortunately, several attempts at MS and crystallization of the RA-V-TAK1 complex failed. Taken together, our data suggest that RA-V blocks the interaction between TAK1 and TAB2 and directly binds the kinase domain of TAK1. Importantly, to our knowledge, this is the first report of a natural product that interrupts the formation of the TAK1–TAB2 complex in the NF-κB pathway. It is worth mentioning that RA-V may inhibit the NF-κB pathway through various ways with different mechanisms, either inside or outside this pathway, and we only focused on the NF-κB signaling pathway itself in this study. In addition, this is the first report to identify TAK1 as RA-V target, which might account for the majority of known bioactivities of RA-V. Besides, the bioactivities against tumor cell proliferation and NF-κB activation of RA-V have good positive correlation in vitro and in vivo, which indicated that RA-V exerts antitumour effect by inhibiting NF-κB activation.

An increasing number of studies have shown that TAK1 plays an essential role in many physiological processes, such as inflammation, tumourigenesis, and metastasis, which makes it an attractive molecular target for the treatment of several human diseases^20^. Recently, researchers found that TAK1 inhibition induces apoptosis in KRAS-dependent colon cancers and promotes NF-κB-dependent cell death in AML cells in vitro and in vivo^45,46^. Therefore, RA-V could be used to develop therapeutic agents that target TAK1. Overall, this study demonstrated that RA-V is a potent new natural inhibitor of the NF-κB signaling pathway, which explains its antiproliferative, antiinflammatory, antiangiogenic and apoptotic effects. In future, we will focus on the role of RA-V in TAK1-associated biological activities or diseases, which will hopefully contribute to its further development as a new clinical remedy for cancer and other related diseases.

## Materials and methods

### Ethics statement

Six- to eight-week-old BALB/c mice and female athymic nude BALB/c mice were purchased from Beijing HFK Bioscience Co., Ltd. (Beijing, China). The mice were maintained in specific pathogen-free conditions at Sichuan University. The animal experiments were performed in strict accordance with the regulations in the guide for the Care and Use of Laboratory Animals of Sichuan University (IACUC number: 20100318).

### Isolation and synthesis of RAs

We isolated natural RAs from the *Rubia* plants *R. yunnanensis*, *R. schumanniana*, *R. cordifolia,* and *R. podantha*^7–9,47,48^. The synthetic RAs were described in our previous study^49^.

### Synthesis and information about the chemical probes

The structures and methods for CB1-12 synthesis are described in Supplementary Figure 3 and the Supplementary Notes.

### Cell lines and culture

HEK293T, HeLa, RAW264.7, HCT116, and HepG2 cell lines were obtained from the American Type Culture Collection (ATCC) and were cultured in Dulbecco’s modified Eagle’s medium (Invitrogen) containing 10% (v/v) heat-inactivated fetal bovine serum (Life Technologies) and 1% (v/v) penicillin–streptomycin (Invitrogen). The cells were maintained at 37°C in a humidified incubator with an atmosphere of 5% CO_2_/95% air (v/v)_._

### Cell transfection and luciferase assay

HEK293T or HeLa cells were seeded in 24-well plates and transiently transfected with the 5 × κB-luciferase and pTK-Renilla reporters using Lipofectamine 2000 (Invitrogen) for 18 h. The cells were then incubated with different concentrations of the compounds for the indicated times and subsequently stimulated with 10 ng/mL TNF-α for 2 h. The luciferase activity in the cell lysates was analyzed using the Dual Luciferase Reporter Assay System (Promega). The luciferase reporter assays were performed as previously described^50^.

### MTT assay

HEK293T cells (5 × 10^3^ cells/well) or HeLa cells (3 × 10^3^ cells/well) were seeded in a 96-well plate with 100 μL medium and cultured for 12 h in a CO_2_ incubator. Then, the cells were treated with various concentrations of RA-V or dimethyl sulfoxide (DMSO) for the indicated times before 3-(4,5-dimethylthiazol-2-yl)-2,5-diphenyltetrazolium bromide (MTT) solution (final concentration at 0.5 mg/mL) was added to each well. After 4 h, the medium was replaced with 150 *μ*L DMSO, and the optical density was measured at 595/650 nM (Molecular Devices). The results are presented as the cell growth inhibition rate.

### Quantitative RT-PCR with reverse transcription

The total RNA was extracted from the indicated cells using TRIzol reagent (Invitrogen) according to the manufacturer’s instructions and then was reverse transcribed. The RNA concentrations were measured spectrophotometrically with a BioPhotometer (Eppendorf). Quantitative real time PCR for *IL-8*, *MCP-1*, *E-selectin*, *CXCL-1*, *IL-6*, *TNF-α*, and *GADPH* was performed with the SYBR Green PCR Master Mix (ABI). The PCR primers used to detect the target genes are listed in Supplementary Table 1.

### Cytokine detection by ELISA

Murine TNF-α and IL-6 were measured using ELISA kits (R&D Systems) according to the manufacturer’s specific instructions.

### Immunofluorescence assay

HeLa cells were plated at a density of 1 × 10^5^ cells per glass coverslip for 24 h before RA-V treatment. After 6 h of incubation with RA-V, the cells were treated with 10 ng/mL TNF-α for 20 min and fixed in 4% paraformaldehyde for 15 min. After permeabilization in 0.1% Triton X-100 for 20 min, the cells were incubated with 5% bovine serum albumin for 1 h, followed by an overnight incubation with an antibody to p65. After three washes in PBS, the coverslips were incubated with a fluorescein isothiocyanate-conjugated goat anti-mouse IgG (Jackson ImmunoResearch) for 1 h. The nuclei were counterstained with 4′,6-diamidino-2-phenylindole (Sigma-Aldrich). The fluorescent signals were examined with a confocal microscope (Leica).

### Immunoprecipitation and western blot analysis

The cell lysates were prepared using radioimmunoprecipitation (RIPA) buffer containing 50 mM Tris-HCl pH 7.4, 150 mM NaCl, 1 mM ethylenediaminetetraacetic acid, 1% Triton X-100, 0.5% deoxycholate, and 0.1% sodium dodecyl sulfate, to which protease inhibitors (Roche) were added. The lysates were incubated with the indicated antibodies before adding 20 μL of Protein A/G agarose for 2 h. After four washes with the same buffer, the immunoprecipitates were boiled in 2 × SDS loading buffer.

For western blot analysis, the samples were equally subjected to sodium dodecyl sulfate polyacrylamide gel electrophoresis (SDS-PAGE) and transferred to polyvinylidene difluoride membranes (Millipore). After blocking with 5% nonfat milk in tris-buffered saline with tween 20, the membranes were incubated with the indicated antibodies for 2 h or overnight at 4°C, followed by an incubation with a horseradish-peroxidase-conjugated secondary antibody for 1 h at room temperature. The protein bands were detected with the SuperSignal West Pico Chemiluminescent Substrate (Pierce).

### Pull-down of RA-V-bound proteins

HEK293T cells were harvested and lysed in RIPA buffer with protease inhibitors. After centrifugation at 12,000 rpm for 15 min, the supernatant was obtained and divided into four equal parts, ensuring that the total protein concentration in each sample was equivalent. The supernatants were precipitated with streptavidin agarose for 1 h before incubation with biotin or CB12 overnight at 4 °C. Then, the beads were incubated with DMSO, biotin or RA-V. After the incubation, the beads were washed four times with RIPA buffer, and the bead-bound proteins were eluted with 2 × SDS loading buffer. The samples were separated by SDS-PAGE, and the bound proteins were detected using the indicated antibodies.

### Preparation of RA-V-loaded polymeric micelles

RA-V-loaded micelles were prepared using thin-film dispersion method. In brief, 10 mg RA-V and 200 mg mPEG2000-PDLLA2000 were completely dissolved in 100 mL mixed solvent (80 mL methylene chloride and 20 mL methanol) to obtain a clear solution in a round-bottom flask. The solvent was removed by rotary evaporation under reduced pressure at 60 °C for about 3 h and a thin layer of uniform film on the wall of the flask was formed. Saline was added to the flask and the mixture was stirred for 10 min at 60 °C to prepare a RA-V-containing polymeric micelle aqueous solution. Then the resultant solution was passed through 0.22 μm filter membrane to remove the unincorporated RA-V aggregates. So the RA-V loaded micelles were obtained.

### Tumor xenograft in nude mice and toxicity assessment

In total, 2 × 10^6^ HCT116 or 3 × 10^6^ HepG2 cells were subcutaneously injected into the right dorsal flank of 6- to 8-week-old female athymic nude BALB/c mice. After tumor volumes reached 100 mm^3^, the tumor-bearing mice were randomly divided into several groups. The mice bearing HCT116 (*n* = 8) or HepG2 xenograft tumors (*n* = 7) were received the intravenous injection of vehicle micelles or various concentrations of RA-V micelles every other day. 5-FU (10 mg/kg) group as positive control. Body weights and tumor volumes were recorded every other day. Tumor volumes were calculated using the following formula: tumor volume (mm^3^) = 0.52 × length × width^2^. At the end of the experiments, the mice were killed. Tumor xenografts of each mouse were removed and weighed. Part of each tumor tissue was stored at liquid nitrogen for quantitative RT-PCR analysis. Organs (heart, kidney, spleen, lung, and liver) from killed mice were also removed, fixed in the 4% formaldehyde solution for microscopic examination by hematoxylin–eosin staining. Serum alanine transaminase(ALT), aspartate transaminase(AST), and creatine kinase(CK) were determined using relevant kits (Nanjing Jiancheng) according to the manufacturer’s specific instructions.

### Bioluminescence imaging

The bioluminescence imaging of the NF-κB-luciferase transgenic mice was performed using a Xenogen IVIS Spectrum in vivo imaging system (Caliper Life Sciences). The mice were anesthetized with isoflurane and intraperitoneally injected with 150 mg/kg D-luciferin in PBS. After 15 min, the in vivo bioluminescence was measured at the indicated times. The bioluminescent signals from all of the mice were detected and quantified.

### Antibody information

The antibodies used included the following: TAK1 (5206, Cell Signaling Technology, dilution 1:2 000), IKKα (11930, Cell Signaling Technology, dilution 1:1 000), IKKβ (8943, Cell Signaling Technology, dilution 1:2 000), TAB2 (3744, Cell Signaling Technology, dilution 1:1 000), IκBα (4812, Cell Signaling Technology, dilution 1:2 000), phospho-TAK1 (4508, Cell Signaling Technology, dilution 1:5 000), phospho-IKKα/β (16A6, Cell Signaling Technology, dilution 1:5 000), phospho-NF-κB p65 (3033, Cell Signaling Technology, dilution 1:8 000), phospho-IκBα (2859, Cell Signaling Technology, dilution 1:2 000), HA (sc-7392, Santa Cruz Biotechnology, dilution 1:2 000), and FLAG (F1804, Sigma, dilution 1: 5 000).

### Plasmids

TAK1 cDNA was obtained from a thymus cDNA library by PCR and subsequently inserted into the pcDNA3.1 vector. The TAK1 truncation mutants were generated from TAK1 cDNA using PCR. The specific primers used for PCR are listed in Supplementary Table 2. The reporter plasmids (5 × κB-luciferase and pTK-Renilla) and the MyD88, TRAF2, TRAF6, TAB1, TAB2, IKKβ, and p65 plasmids have been described previously^51^. All of the plasmids were analytically verified by sequencing.

### Purchased chemical compounds

The chemicals and solvents used to synthesize the derivatives and probes were purchased from J&K or Aladdin. mPEG2000-PDLLA2000 was obtained from Jinan Daigang Biomaterial Co.,Ltd.

### Molecular modeling

The Auto Dock vina docking program^52^ was used to predict the RA-V binding site on the TAK1 protein. The crystal structure of TAK1 (TAK1–TAB1 complex, PDB: 4GS6) was used as the receptor, and the RA-V structure modified from the crystal structure of RA-XXIII-Br (CCDC: 672443) was used as the ligand. The model of the RA-V-TAK1 complex was also used to generate the pharmacophore for the pharmacophore screen using the Discovery Studio 4.0 package.

### DrugCIPHER

This computational method was performed as previously described^27^.

### Statistical analysis

The Student’s *t* test was used for the statistical analysis. A *p* value < 0.05 was considered statistically significant. The statistical analysis was performed using GraphPad Prism software.

## Electronic supplementary material

Supplementary Information
