# Supplementary material for: Natural cyclopeptide RA-V inhibits the NF-κB signaling pathway by targeting TAK1
Source: Cell Death Dis. 2018 Jun 18;9(7):715. doi: 10.1038/s41419-018-0743-2 (PMC6006164; doi:10.1038/s41419-018-0743-2)
Supplement: Supplementary file 1 — Supplementary Information [file 41419_2018_743_MOESM1_ESM.pdf]

Supplementary Information

Natural cyclopeptide RA-V inhibits the NF-κB signaling pathway by targeting TAK1

Zhe Wang[1,2,*], Simeng Zhao[2,*], Lihua Song[1,*], Yuzhi Pu[3], Qiang Wang[4], Guangzhi Zeng[2], Xing Liu[4], Ming Bai[5], Shao Li[5], Fabao Gao[3], Lijuan Chen[3,**], Chen Wang[1,4,**] & Ninghua Tan[1,2,**]

[1] School of Traditional Chinese Pharmacy & State Key Laboratory of Natural Medicines, China Pharmaceutical University, Nanjing 211198, PR China;

[2] State Key Laboratory of Phytochemistry and Plant Resources in West China, Kunming Institute of Botany, Chinese Academy of Sciences, Kunming 650201 China;

[3] State Key Laboratory of Biotherapy / Collaborative Innovation Center of Biotherapy and Cancer Center, West China Hospital of Sichuan University, Chengdu 610041, China；

[4] State Key Laboratory of Cell Biology, Shanghai Institute of Biochemistry and Cell Biology, Chinese Academy of Sciences, Shanghai 200031, China;

[5] MOE Key Laboratory of Bioinformatics and Bioinformatics Division, TNLIST / Department of Automation, Tsinghua University, Beijing 100084, PR China;

***** These authors contributed equally to this work.

****** Correspondence and requests for materials should be addressed to: L.J.C (email: chenlijuan125@163.com) or to C.W (email: cwang1971@cpu.edu.cn) or to N.H.T (email: nhtan@cpu.edu.cn).

Ⅰ. Supplementary Results

Supplementary Figure 1. RA-V has the best inhibitory effect on NF-κB signaling pathway among RAs. (a-b) HEK293T cells were transfected with the 5×κB-luciferase and pTK-Renilla reporters. Twenty-four hours after transfection, the cells were incubated with various concentrations of the indicated compounds for 6 h and then treated with 10 ng/ml TNF-α for 2 h before the luciferase activity assays. The data are presented as the means ± S.D. from three independent experiments.

Supplementary Figure 2. RA-V exerts cytotoxic activity in HEK293T and HeLa cells. HEK293T or HeLa cells were incubated with various concentrations of RA-V for the indicated times and then analyzed by MTT assay. Taxol was used as the positive control, and the data are presented as the means ± S.D. from three independent experiments.

Supplementary Figure 3. Identification of potential RA-V targets using the chemical probe CB12. (a) The chemical structures of CB1-12. (b) Scheme for CB12 synthesis. (c) CB12 inhibited NF-κB reporter activity in a dose-dependent manner. The HEK293T cells were transfected with the 5×κB-luciferase and pTK-Renilla reporters. Twenty-four h after transfection, the cells were treated with various concentrations of CB12 for 6 h and then treated with 10 ng/ml TNF-α for 2 h before the luciferase activity assays. (d) HEK293T cells were treated with various concentrations of CB12 for the indicated times and then analyzed with an MTT assay. The data are presented as the means ± S.D. from three independent experiments.

Supplementary Figure 4. The pharmacophore screen for RAs. (a) The pharmacophore generated by the model of the RA-V-TAK1 complex (Green:

HB acceptor; Magenta: HB donor; Cyan: Hydrophobic; Grey: Excluded volume). (b) The fit values for the RAs match the bioactivities well. A complete screen of the RAs with the generated pharmacophore was performed using the Discovery Studio 4.0 package. The fit values gather the bioactive compounds, which indicates the correctness of the predicted model.

a

NO.	Compound	IC_{50} (μM)	NO.	Compound	IC_{50} (μM)	NO.	Compound	IC_{50} (μM)
1	RA-V	0.065	13	RA-XI isoform 2	ND	25	C-6β-oxy-RA-IV	2.06
2	RA-XI isoform 1	ND	14	Rubiyunnanin F	ND	26	Rubicordifolin A	3.41
3	RA-XXIV	11.86	15	Rubiyunnanin B	ND	27	Rubicordifolin B	NA
4	RA-XII	1.85	16	Rubiyunnanin C	ND	28	Rubicordifolin C	6.87
5	RA-I	1.31	17	Rubiyunnanin D	ND	29	RA-III	1.1
6	Rubiyunnanin A	ND	18	Rubiyunnanin E	ND	30	5[a]	ND
7	RY-II	ND	19	RA-VII	0.089	31	4[a]	ND
8	Rubiyunnanin G	ND	20	Rubiaschumanin A	ND	32	6[a]	ND
9	Rubiyunnanin I	ND	21	Rubiaschumanin C	ND	33	7[a]	ND
10	Rubiyunnanin H	ND	22	Rubiaschumanin B	3.88	34	8[a]	ND
11	RA-XIII	ND	23	O-seco-RA-V	ND			
12	RA-XIII-OMe	ND	24	RA-IV	10.59			

ND: no detected (IC_{50} >15 μM).

a: compound 4-8 in reference 47.

b

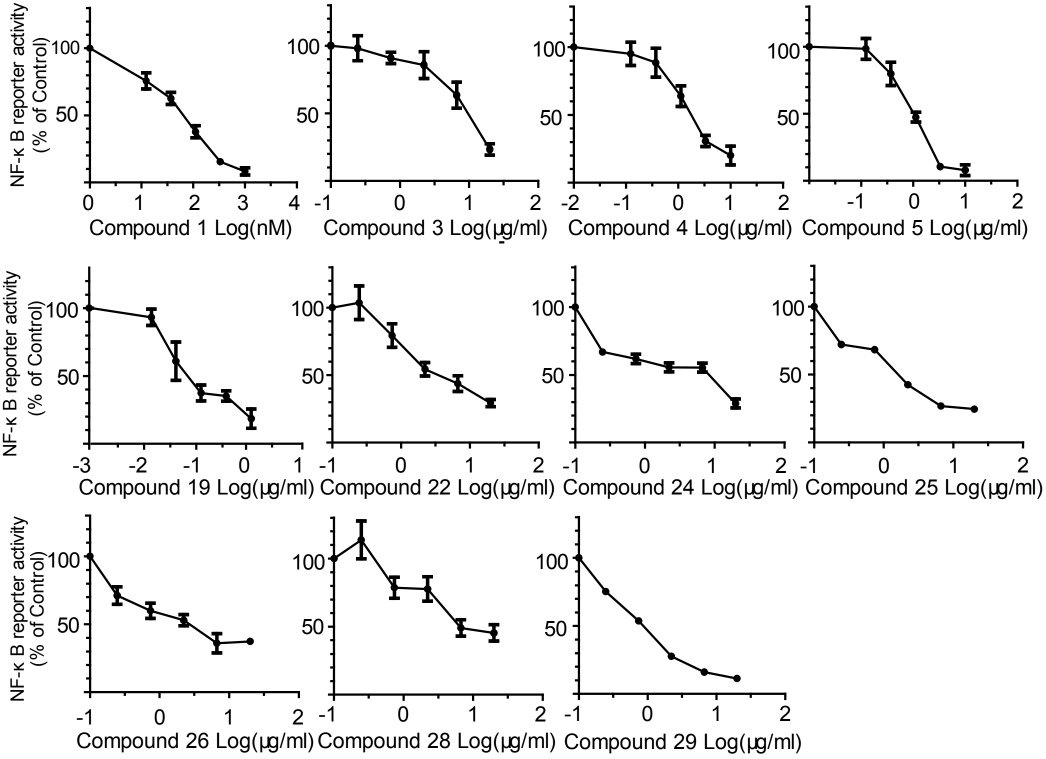

Supplementary Figure 1. RA-V has the best inhibitory effect on NF-κB signaling pathway among RAs.

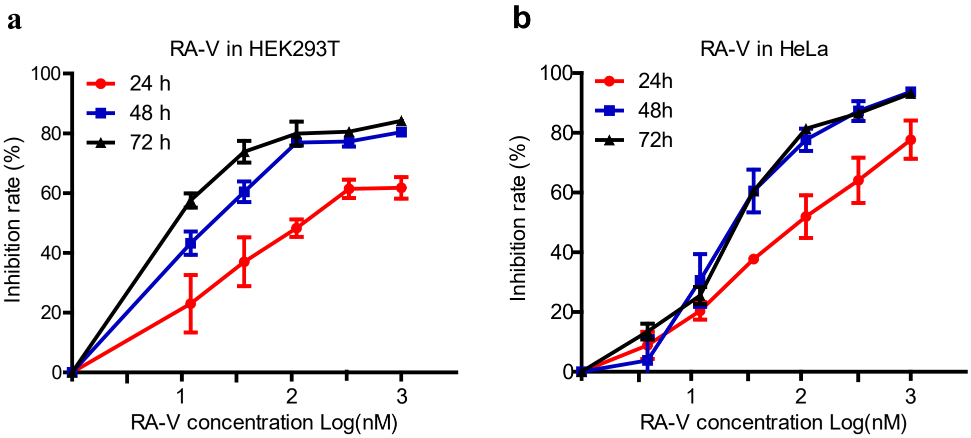

Supplementary Figure 2. RA-V exerts cytotoxic activity in HEK293T and HeLa cells.

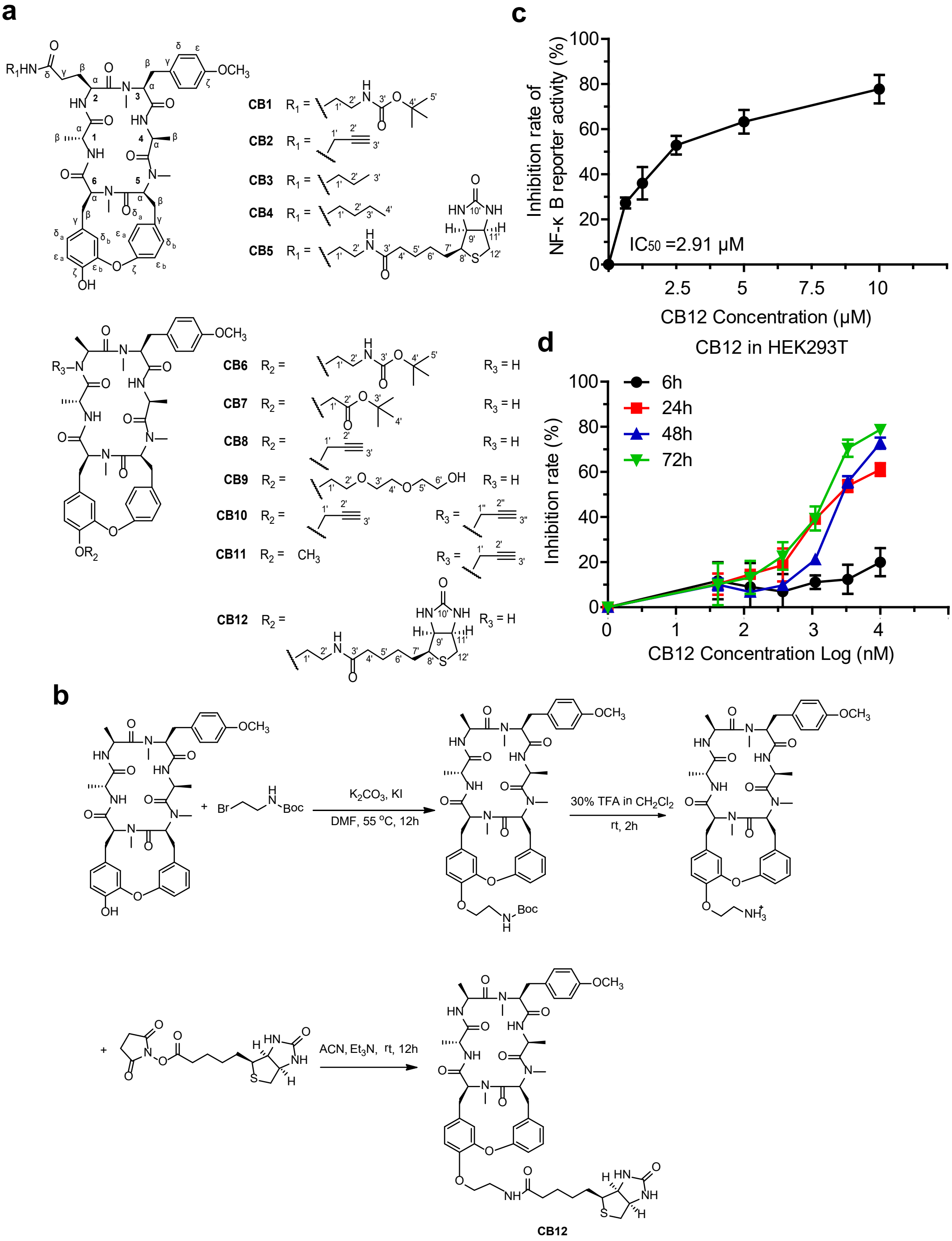

Supplementary Figure 3. Identification of potential RA-V targets using the chemical probe CB12.

a

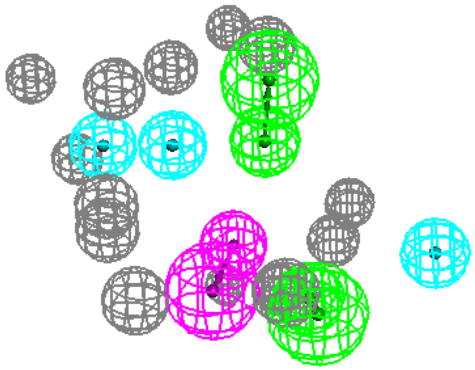

b

NO.	Name	IC_{50} (μM)	Fit value
1	RA-V	0.065	2.719
19	RA-VII	0.089	2.719
4	RA-XII	1.85	2.719
10	Rubiyunnanin H	ND	2.719
24	RA-IV	10.59	2.719
25	6-β-oxy-RA-IV	2.06	1.824
15	Rubiyunnanin B	15.07	1.293
22	Rubischumanin B	3.88	1.293
20	Rubischumanin A	ND	0.892
7	RY-II	18.97	0.888
30	5[a]	ND	0.847
32	6[a]	ND	0.746
29	RA-III	1.1	0.72
5	RA-I	1.31	0.667
28	Rubicordifolin C	6.87	0.01
23	O-seco-RA-V	ND	0.005

a: compound 4-8 in reference 48.

Supplementary Figure 4. The pharmacophore screening of RAs.

Ⅱ. Supplementary Tables for Methods

Supplementary Table 1: Primers used for quantitative RT-PCR assays

Genes	Forward primer (5' - 3')	Reverse primer (5' - 3')
IL-8 **(Homo sapiens)**	GGCAGCCTTCCTGATTTCTG	CTTGGCAAAACTGCACCTTCA
MCP-1 **(Homo sapiens)**	TCGCCTCCAGCATGAAAGTC	GGCATTGATTGCATCTGGC
E-selectin **(Homo sapiens)**	CTGCCTGTACCAATACATCC	CAGTTCACAATTTGCTCACAC
GADPH **(Homo sapiens)**	CGGAGTCAACGGATTTGGTC	GACAAGCTTCCCGTTCTCAG
IL-6 (mus musculus)	GAGAGGAGACTTCACAGAGGATAC	GTACTCCAGAAGACCAGAGG
TNF-α (mus musculus)	CATCTTCTCAAAATTCGAGTGACAA	CCAGCTGCTCCTCCACTTG
GADPH (mus musculus)	GAAGGGCTCATGACCACAGT	GGATGCAGGGATGATGTTCT

Supplementary Table 2: Primers for TAK1 constructs

Genes	Primers: Forward primers (F), Reverse primers (R)
**TAK1 (Homo sapiens)**	F: AATGAATTCATGTCTACAGCCTCTGCCGC<br>R: AATCTCGAGTCATGAAGTGCCTTGTCGTT
**TAK1 (1-303) (Homo sapiens)**	F: AATGAATTCATGTCTACAGCCTCTGCCGC<br>R: GGCCTCGAGCTACTGACAAGGATACTGTA
**TAK1 (304-579) (Homo sapiens)**	F: GGCGAATTCTATTCAGATGAAGGACAGAG<br>R: AATCTCGAGTCATGAAGTGCCTTGTCGTT

Supplementary Table 3: Screening data of small molecules

Category	Parameter	Description
Assay	Type of assay	Cell-based
	Target	NF-κB signaling pathway
	Primary measurement	Detection of Firefly luciferase enzyme activities and Renilla Luciferase expression
	Key reagents	5 × κB-lucieferase and luciferase assay (Progema)
	Assay protocol	HEK293T cells were seeded in 24-well plates and transiently transfected with 20 ng of 5×κB-luciferase and 2 ng of pTK-Renilla reporters using Lipofectamine 2000 (Invitrogen) for 18 h. The cells were then incubated with small molecules (10 μM) for 6 hours, and subsequently stimulated with 10 ng/ml TNF-α for 2 h. Luciferase activity of the cell lysates was analysed by the Dual Luciferase Reported Assay System (Promega). The Firefly luciferase enzyme activities were determined for NF-κB signaling activities and normalized to Renilla Luciferase expression.
	Additional comments	no
Library	Library size	approximately 200
	Library composition	natural and synthetic chemical compounds, including cyclopeptides, quinones and terpenes.
	Source	State Key Laboratory of Phytochemistry and Plant Resources in West China, Kunming Institute of Botany, Chinese Academy of Sciences
	Additional comments	no
Screen	Format	24-well, Corning
	Concentration(s) tested	10 μM
	Plate controls	Velcade
	Reagent/ compound dispensing system	Manual
	Detection instrument and software	Synergy 2 (BioTek)
	Assay validation/QC	Velcade inhibits NF-κB signaling pathway
	Correction factors	no
	Normalization	The Firefly luciferase enzyme activities were normalized by Renilla Luciferase expression
	Additional comments	no
Post-HTS analysis	Hit criteria	κB-luc/ Renilla ratio < 0.5 standard deviations from the mean
	Hit rate	Approximately 15%
	Additional assay(s)	no
	Confirmation of hit purity and structure	Compounds were verified by HPLC and NMR
	Additional comments	no

III. Supplementary Notes

Synthesis and information of chemical probes

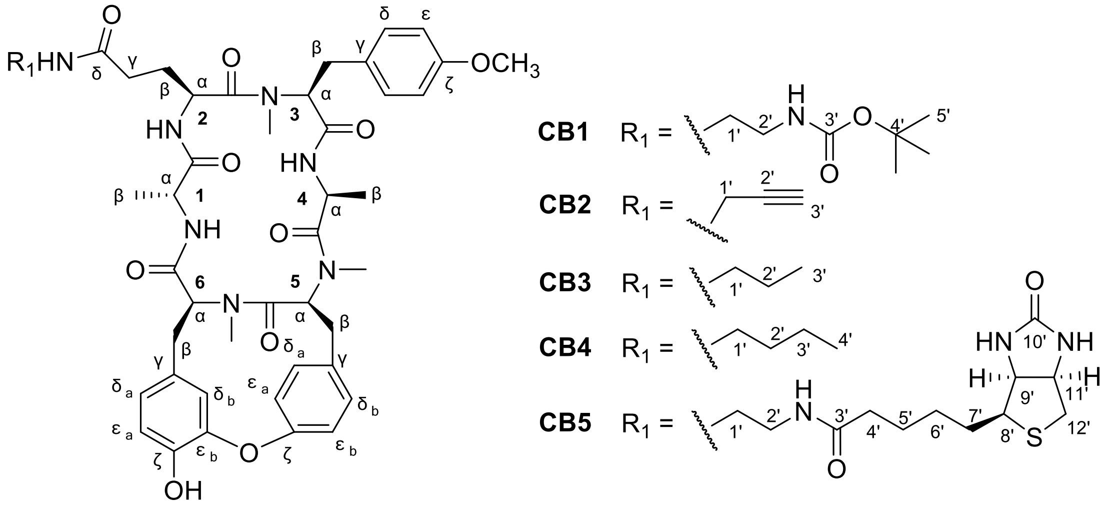

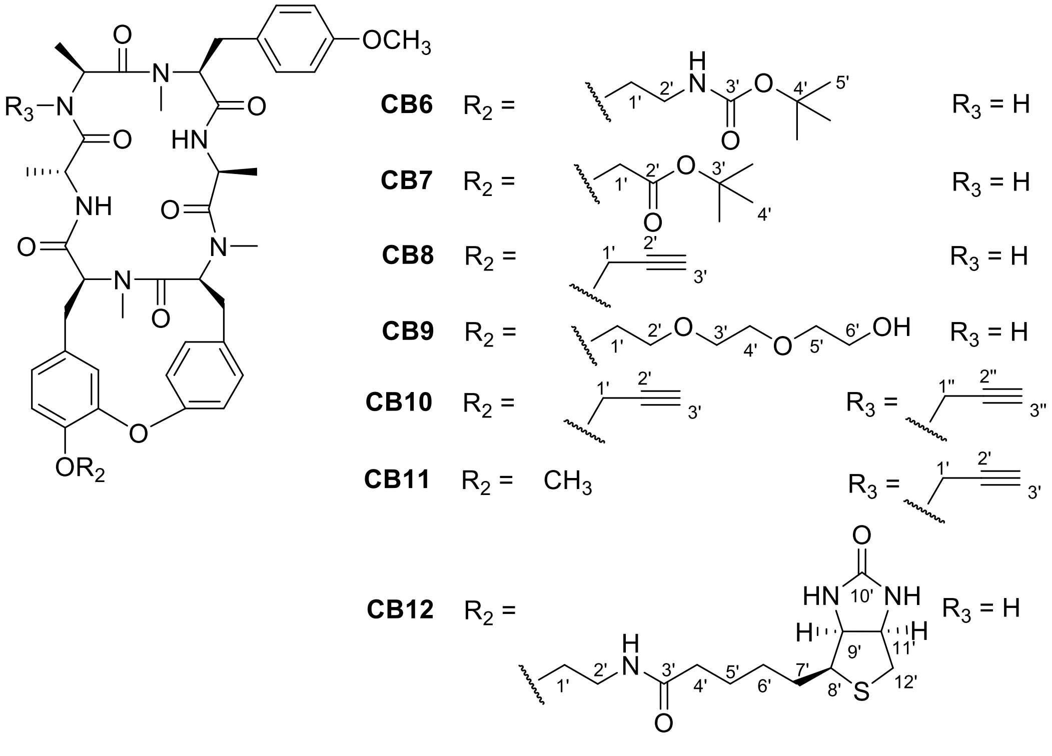

CB1: The mixture of RA-XI (16.0 mg, 0.020 mmol), HATU (17.3 mg, 0.046 mmol), and HOAt (5.8 mg, 0.043 mmol) were dissolved in DMF (1.0 mL), following N-Boc-ethylenediamine (6.0 μL, 0.04 mmol) and Et_3N (20.0 μL) were added and stirred at room temperature for 12 h. The reaction solution was then directly purified by HPLC (35 % ACN-65 % H_2O) to yield compound **CB1** as

white amorphous powder (15.5 mg, 83 %).

CB1: white amorphous powder; $C_{49}H_{64}N_8O_{12}$; ^{1}H NMR (C_5D_5N, 400 MHz): δ 5.15 (1H, overlap, H-1α), 1.48 (3H, d, J = 7.0 Hz, H-1β), 8.77 (1H, d, J = 8.5 Hz, 1-NH), 5.42 (1H, m, H-2α), 2.58 (2H, m, H-2β), 2.62 (2H, m, H-2γ), 9.83 (1H, d, J = 8.0 Hz, 2-NH), 4.08 (1H, t, J = 7.0 Hz, H-3α), 3.85 (2H, d, J = 7.0 Hz, H-3β), 7.31 (2H, d, J = 8.5 Hz, H-3δ), 6.98 (2H, d, J = 8.5 Hz, H-3ε), 3.27 (3H, s, 3-NCH_3), 3.64 (3H, s, 3-OCH_3), 5.05 (1H, overlap, H-4α), 1.35 (3H, d, J = 6.0 Hz, H-4β), 7.36 (d, J = 7.0 Hz, 4-NH), 5.75 (1H, dd, J = 11.0, 3.5 Hz, H-5α), 2.62 (1H, overlap, H-5βa), 3.65 (1H, overlap, H-5βb), 7.44 (1H, dd, J = 8.5, 2.0 Hz, H-5δa), 7.21 (1H, overlap, H-5δb), 6.90 (1H, dd, J = 8.5, 2.0 Hz, H-5εa), 7.21 (1H, overlap, H-5εb), 3.00 (3H, s, 5-NCH_3), 5.03 (1H, dd, J = 12.0, 3.0 Hz, H-6α), 3.57 (1H, overlap, H-6βa), 3.34 (1H, m, H-6βb), 6.76 (1H, d, J = 7.5 Hz, H-6δa), 4.64 (1H, br s, H-6δb), 6.94 (1H, d, J = 8.5 Hz, H-6εa), 3.02 (3H, s, 6-NCH_3), 2.59 (2H, overlap, H-1'), 2.59 (2H, overlap, H-2'),1.44 (9H, s, H-5'); ^{13}C NMR (C_5D_5N, 100 MHz): δ 48.3 (d, C-1α), 21.7 (q, C-1β), 172.9 (s, 1-CO), 48.8 (d, C-2α), 27.5 (t, C-2β), 32.1 (t, C-2γ), 172.5 (s, C-2δ), 173.0 (s, 2-CO), 68.8 (d, C-3α), 33.9 (t, C-3β), 132.0 (s, C-3γ), 130.9 (d, C-3δ), 114.5 (d, C-3ε), 158.8 (s, C-3ζ), 168.7 (s, 3-CO), 40.1 (q, 3-NCH_3), 55.2 (q, 3-OCH_3), 46.9 (d, C-4α), 19.2 (q, C-4β), 172.1 (s, 4-CO), 54.5 (d, C-5α), 36.8 (t, C-5β), 136.1 (s, C-5γ), 133.6 (d, C-5δa), 131.0 (d, C-5δb), 124.7 (d, C-5εa), 126.6 (d, C-5εb), 158.8 (s, C-5ζ), 169.9 (s, 5-CO), 30.4 (q, 5-NCH_3), 57.9 (d, C-6α), 36.3 (t, C-6β), 128.1 (s, C-6γ), 122.1 (d, C-6δa), 115.1 (d, C-6δb), 117.9 (d, C-6εa), 152.8 (s, C-6εb), 145.5 (s, C-6ζ), 171.3 (s, 6-CO), 29.8 (q, 6-NCH_3), 41.0 (t, C-1'), 40.2 (t, C-2'), 157.0 (s, C-3'), 78.3 (s, C-4'), 28.6 (q, C-5'); ESIMS (positive): *m/z* 979 $[M+Na]^+$; HRESIMS: *m/z* 979.4548 (calcd for

$C_{49}H_{64}N_8NaO_{12}$, 979.4541).

CB2: The mixture of RA-XI (16.4 mg, 0.020 mmol), HATU (20.3 mg, 0.053 mmol), and HOAt (11.9 mg, 0.088 mmol) were dissolved in DMF (1.0 mL), following 2-propynylamine (6.0 *μ*L, 0.09 mmol) and Et_3N (30.0 *μ*L) were added and stirred at room temperature for 12 h. The reaction solution was then directly purified by HPLC (35 % ACN-65 % H_2O) to yield compound **CB2** as white amorphous powder (16.2 mg, 96 %).

CB2: white amorphous powder; $C_{45}H_{53}N_7O_{10}$; ^1H NMR ($CDCl_3$, 400 MHz): *δ* 4.71 (1H, overlap, H-1α), 1.27 (3H, d, *J* = 6.5 Hz, H-1β), 6.74 (1H, d, *J* = 8.5 Hz, 1-NH), 4.43 (1H, m, H-2α), 2.02 (2H, m, H-2β), 2.59 (2H, overlap, H-2γ), 3.62 (1H, m, H-3α), 3.37 (1H, dd, *J* = 13.5, 3.5 Hz, H-3βa), 3.27 (1H, dd, *J* = 13.5, 11.0 Hz, H-3βb), 7.03 (2H, d, *J* = 8.0 Hz, H-3δ), 6.82 (2H, d, *J* = 8.0 Hz, H-3ε), 2.92 (3H, s, 3-NCH_3), 3.76 (3H, s, 3-OCH_3), 4.71 (1H, overlap, H-4α), 1.03 (3H, d, *J* = 6.0 Hz, H-4β), 6.47 (d, *J* = 7.0 Hz, 4-NH), 5.37 (1H, dd, *J* = 11.0, 2.0 Hz, H-5α), 2.63 (1H, overlap, H-5βa), 3.66 (1H, overlap, H-5βb), 7.23 (1H, overlap, H-5δa), 7.38 (1H, dd, *J* = 8.5, 2.0 Hz, H-5δb), 6.83 (1H, overlap, H-5εa), 7.16 (1H, dd, *J* = 8.5, 2.0 Hz, H-5εb), 2.93 (3H, s, 5-NCH_3), 4.50 (1H, dd, *J* = 11.5, 3.5 Hz, H-6α), 3.03 (1H, overlap, H-6βa), 2.96 (1H, overlap, H-6βb), 6.50 (1H, d, *J* = 7.5 Hz, H-6δa), 4.30 (1H, br s, H-6δb), 6.80 (1H, overlap, H-6εa), 2.65 (3H, s, 6-NCH_3), 2.85 (2H, s, H-1'), 3.05 (1H, s, H-3'); ^{13}C NMR ($CDCl_3$, 100 MHz): *δ* 46.3 (d, C-1α), 20.6 (q, C-1β), 171.6 (s, 1-CO), 48.8 (d, C-2α), 26.0 (t, C-2β), 31.7 (t, C-2γ), 171.8 (s, C-2δ), 172.6 (s, 2-CO), 68.2 (d, C-3α), 32.9 (t, C-3β), 130.5 (s, C-3γ), 130.1 (d, C-3δ), 114.1 (d, C-3ε), 158.4 (s, C-3ζ), 168.2 (s, 3-CO), 40.1 (q, 3-NCH_3), 55.3 (q, 3-OCH_3), 47.8 (d, C-4α), 18.5 (q, C-4β), 171.6 (s, 4-CO), 54.3 (d, C-5α), 36.8 (t, C-5β), 135.6 (s,

C-5γ), 132.9 (d, C-5δa), 131.0 (d, C-5δb), 124.1 (d, C-5εa), 125.9 (d, C-5εb), 157.9 (s, C-5ζ), 169.1 (s, 5-CO), 30.5 (q, 5-NCH_3), 57.3 (d, C-6α), 35.5 (t, C-6β), 127.5 (s, C-6γ), 121.6 (d, C-6δa), 113.0 (d, C-6δb), 115.8 (d, C-6εa), 151.1 (s, C-6εb), 143.0 (s, C-6ζ), 170.6 (s, 6-CO), 29.4 (q, 6-NCH_3), 29.1 (t, C-1'), 79.5 (s, C-2'), 71.5 (d, C-3'); ESIMS (positive): *m/z* 874 $[M+Na]^+$; HRESIMS: *m/z* 874.3742 (calcd for $C_{45}H_{53}N_7NaO_{10}$, 874.3752).

CB3: The mixture of RA-XI (10.3 mg, 0.013 mmol), HATU (12.9 mg, 0.034 mmol), and HOAt (5.4 mg, 0.040 mmol) were dissolved in DMF (1.0 mL), following propylamine (10.0 μL, 0.122 mmol) and Et_3N (30.0 μL) were added and stirred at room temperature for 12 h. The reaction solution was then directly purified by HPLC (35 % ACN-65 % H_2O) to yield compound **CB3** as white amorphous powder (8.0 mg, 77 %).

CB3: white amorphous powder; $C_{45}H_{57}N_7O_{10}$; ^{1}H NMR ($CDCl_3$, 400 MHz): δ 4.65 (1H, m, H-1α), 1.18 (3H, d, *J* = 6.5 Hz, H-1β), 4.30 (1H, m, H-2α), 2.02 (2H, m, H-2β), 2.56 (2H, overlap, H-2γ), 3.60 (1H, overlap, H-3α), 3.29 (1H, dd, *J* = 13.5, 4.0 Hz, H-3βa), 3.20 (1H, dd, *J* = 13.5, 10.5 Hz, H-3βb), 6.99 (2H, d, *J* = 8.0 Hz, H-3δ), 6.76 (2H, d, *J* = 8.0 Hz, H-3ε), 2.87 (3H, s, 3-NCH_3), 3.70 (3H, s, 3-OCH_3), 4.65 (1H, overlap, H-4α), 0.98 (3H, d, *J* = 6.5 Hz, H-4β), 5.30 (1H, dd, *J* = 11.0, 2.5 Hz, H-5α), 2.56 (1H, overlap, H-5βa), 3.57 (1H, overlap, H-5βb), 7.14 (1H, dd, *J* = 8.5, 2.0 Hz, H-5δa), 7.32 (1H, dd, *J* = 8.5, 2.0 Hz, H-5δb), 6.78 (1H, overlap, H-5εa), 7.12 (1H, dd, *J* = 8.5, 2.0 Hz, H-5εb), 2.99 (3H, s, 5-NCH_3), 4.49 (1H, dd, *J* = 11.0, 3.5 Hz, H-6α), 2.99 (1H, overlap, H-6βa), 2.91 (1H, overlap, H-6βb), 6.41 (1H, dd, *J* = 8.5, 2.0 Hz, H-6δa), 4.26 (1H, br s, H-6δb), 6.68 (1H, d, *J* = 8.5 Hz, H-6εa), 2.58 (3H, s, 6-NCH_3), 3.02 (2H, overlap, H-1'), 1.40 (2H, m, H-2'), 0.81 (3H, d, *J* = 7.5 Hz, H-3'); ^{13}C NMR

($CDCl_3$, 100 MHz): *δ* 46.3 (d, C-1α), 20.4 (q, C-1β), 171.6 (s, 1-CO), 48.4 (d, C-2α), 26.3 (t, C-2β), 31.7 (t, C-2γ), 171.9 (s, C-2δ), 172.4 (s, 2-CO), 68.2 (d, C-3α), 32.8 (t, C-3β), 130.5 (s, C-3γ), 130.1 (d, C-3δ), 114.1 (d, C-3ε), 158.4 (s, C-3ζ), 168.4 (s, 3-CO), 39.9 (q, 3-NCH_3), 55.2 (q, 3-OCH_3), 47.6 (d, C-4α), 18.0 (q, C-4β), 171.6 (s, 4-CO), 54.4 (d, C-5α), 36.7 (t, C-5β), 135.2 (s, C-5γ), 132.7 (d, C-5δa), 130.7 (d, C-5δb), 124.1 (d, C-5εa), 125.9 (d, C-5εb), 158.2 (s, C-5ζ), 169.4 (s, 5-CO), 30.4 (q, 5-NCH_3), 57.4 (d, C-6α), 35.5 (t, C-6β), 127.2 (s, C-6γ), 121.4 (d, C-6δa), 113.4 (d, C-6δb), 116.2 (d, C-6εa), 151.4 (s, C-6εb), 143.3 (s, C-6ζ), 170.8 (s, 6-CO), 29.3 (q, 6-NCH_3), 41.2 (t, C-1'), 22.5 (t, C-2'), 11.1 (q, C-3'); ESIMS (positive): *m/z* 878 $[M+Na]^+$; HRESIMS: *m/z* 878.4054 (calcd for $C_{45}H_{57}N_7NaO_{10}$, 878.4065).

CB4: The mixture of RA-XI (11.7 mg, 0.014 mmol), HATU (14.0 mg, 0.030 mmol), and HOAt (6.1 mg, 0.045 mmol) were dissolved in DMF (1.0 mL), following butylamine (10.0 *μ*L, 0.103 mmol) and Et_3N (30.0 *μ*L) were added and stirred at room temperature for 12 h. The reaction solution was then directly purified by HPLC (30 % ACN-70 % H_2O) to yield compound **CB4** as white amorphous powder (7.0 mg, 56 %).

CB4: white amorphous powder; $C_{46}H_{49}N_7O_{10}$; ^1H NMR ($CDCl_3$, 400 MHz): *δ* 4.67 (1H, m, H-1α), 1.20 (3H, d, *J* = 7.0 Hz, H-1β), 4.31 (1H, m, H-2α), 1.92 (2H, m, H-2β), 2.56 (2H, m, H-2γ), 3.59 (1H, dd, *J* = 10.5, 4.0 Hz, H-3α), 3.29 (1H, overlap, H-3βa), 3.23 (1H, dd, *J* = 14.0, 10.5 Hz, H-3βb), 7.01 (2H, d, *J* = 8.5 Hz, H-3δ), 6.79 (2H, d, *J* = 8.5 Hz, H-3ε), 2.89 (3H, s, 3-NCH_3), 3.72 (3H, s, 3-OCH_3), 4.60 (1H, m, H-4α), 1.01 (3H, d, *J* = 6.5 Hz, H-4β), 5.33 (1H, dd, *J* = 11.5, 3.0 Hz, H-5α), 2.56 (1H, overlap, H-5βa), 3.57 (1H, overlap, H-5βb), 7.17 (1H, dd, *J* = 8.5, 2.0 Hz, H-5δa), 7.34 (1H, dd, *J* = 8.0, 2.0 Hz, H-5δb), 6.79 (1H,

overlap, H-5εa), 7.14 (1H, dd, J = 8.0, 2.0 Hz, H-5εb), 3.02 (3H, s, 5-NCH_3), 4.51 (1H, dd, J = 12.0, 3.5 Hz, H-6α), 2.99 (1H, overlap, H-6βa), 2.91 (1H, overlap, H-6βb), 6.43 (1H, dd, J = 8.0, 2.0 Hz, H-6δa), 4.29 (1H, br s, H-6δb), 6.71 (1H, d, J = 8.0 Hz, H-6εa), 2.60 (3H, s, 6-NCH_3), 3.04 (2H, overlap, H-1'), 1.37 (2H, m, H-2'), 1.22 (2H, overlap, H-3'), 0.84 (3H, d, J = 7.0 Hz, H-4'); ^{13}C NMR ($CDCl_3$, 100 MHz): δ 46.3 (d, C-1α), 20.4 (q, C-1β), 171.6 (s, 1-CO), 48.5 (d, C-2α), 26.4 (t, C-2β), 31.8 (t, C-2γ), 171.9 (s, C-2δ), 172.4 (s, 2-CO), 68.2 (d, C-3α), 32.9 (t, C-3β), 130.6 (s, C-3γ), 130.1 (d, C-3δ), 114.1 (d, C-3ε), 158.4 (s, C-3ζ), 168.3 (s, 3-CO), 39.9 (q, 3-NCH_3), 55.2 (q, 3-OCH_3), 47.6 (d, C-4α), 18.1 (q, C-4β), 171.6 (s, 4-CO), 54.4 (d, C-5α), 36.7 (t, C-5β), 135.3 (s, C-5γ), 132.8 (d, C-5δa), 130.8 (d, C-5δb), 124.1 (d, C-5εa), 125.9 (d, C-5εb), 158.2 (s, C-5ζ), 169.3 (s, 5-CO), 30.4 (q, 5-NCH_3), 57.4 (d, C-6α), 35.5 (t, C-6β), 127.3 (s, C-6γ), 121.5 (d, C-6δa), 113.3 (d, C-6δb), 116.1 (d, C-6εa), 151.3 (s, C-6εb), 143.2 (s, C-6ζ), 170.8 (s, 6-CO), 29.3 (q, 6-NCH_3), 39.3 (t, C-1'), 31.4 (t, C-2'), 19.9 (t, C-3'), 13.5 (q, C-4'); ESIMS (positive): m/z 892 $[M+Na]^+$; HRESIMS: m/z 892.4211 (calcd for $C_{46}H_{59}N_7NaO_{10}$, 892.4221).

CB5: To the solution of Biotin-NHS (978.4 mg, 2.869 mmol) in DMF (25 mL), N-Boc-ethylenediamine (650.0 μL, 4.128 mmol) and Et_3N (900.0 μL) were added and stirred at room temperature for 12 h. The reaction solution was subjected to a RP-18 column and subsequently diluted by 5 % MeOH and then by 95 % MeOH, the 95 % MeOH fraction was collected and evaporated to yield biotinylated N-Boc-ethylenediamine as white amorphous powder (1052 mg, 95 %); Biotinylated N-Boc-ethylenediamine (100.4 mg, 0.260 mmol) was then dissolved in HCl-dioxane (5 mL, 25 %) and stirred for 12 h at room temperature. After the solution was evaporated, RA-XI (62.6 mg, 0.077 mmol),

HATU (58.6 mg, 0.154 mmol), and HOAt (21.0 mg, 0.154 mmol) were added and dissolved in DMF (5.0 mL), Et_3N (80 *μ*L) was then added and further stirred at room temperature for 12 h. The reaction solution was purified by HPLC (70 % MeOH-30 % H_2O) to yield compound **CB5** as white amorphous powder (47.0 mg, 56 %).

CB5: white amorphous powder; $C_{54}H_{70}N_{10}O_{12}S$; ^{1}H NMR (DMSO-$d_6$, 500 MHz): *δ* 4.63 (1H, overlap, H-1α), 1.06 (3H, d, *J* = 7.0 Hz, H-1β), 4.48 (1H, m, H-2α), 1.79 (2H, m, H-2β), 2.05 (2H, overlap, H-2γ), 8.44 (1H, d, *J* = 7.0 Hz, 2-NH), 3.88 (1H, m, H-3α), 3.39 (1H, overlap, H-3βa), 3.05 (1H, overlap, H-3βb), 7.09 (2H, d, *J* = 8.0 Hz, H-3δ), 6.84 (2H, d, *J* = 8.0 Hz, H-3ε), 2.92 (3H, s, 3-NCH_3), 3.71 (3H, s, 3-OCH_3), 4.58 (1H, m, H-4α), 0.92 (3H, d, *J* = 6.5 Hz, H-4β), 6.60 (d, *J* = 8.0 Hz, 4-NH), 5.31 (1H, d, *J* = 11.0 Hz, H-5α), 2.69 (1H, overlap, H-5βa), 3.46 (1H, overlap, H-5βb), 7.26 (1H, d, *J* = 8.0 Hz, H-5δa), 7.40 (1H, dd, *J* = 8.0, 1.0 Hz, H-5δb), 6.88 (1H, d, *J* = 8.0 Hz, H-5εa), 6.66 (1H, d, *J* = 8.0 Hz, H-5εb), 2.88 (3H, s, 5-NCH_3), 4.60 (1H, m, H-6α), 3.13 (1H, overlap, H-6βa), 3.05 (1H, overlap, H-6βb), 6.44 (1H, d, *J* = 8.0 Hz, H-6δa), 4.46 (1H, br s, H-6δb), 6.74 (1H, d, *J* = 8.0 Hz, H-6εa), 2.46 (3H, s, 6-NCH_3), 3.00 (2H, overlap, H-1'), 3.00 (2H, overlap, H-2'), 2.03 (2H, overlap, H-4'), 1.47 (2H, m, H-5'), 1.26 (2H, m, H-6'), 1.58 (2H, m, H-7'), 3.06 (1H, overlap, H-8'), 4.11 (1H, m, H-9'), 4.28 (1H, m, H-11'), 2.78 (1H, overlap, H-12'a), 2.56 (1H, d, *J* = 12.5 Hz, H-12'b), 7.82 (1H, d, *J* = 5.0 Hz, 2'-NH), 6.36 (1H, s, 9'-NH), 6.43 (1H, s, 11'-NH); ^{13}C NMR (DMSO-d_6, 125 MHz): *δ* 45.5 (d, C-1α), 21.1 (q, C-1β), 171.5 (s, 1-CO), 47.6 (d, C-2α), 25.2 (t, C-2β), 29.1 (t, C-2γ), 171.6 (s, C-2δ), 172.3 (s, 2-CO), 66.7 (d, C-3α), 31.6 (t, C-3β), 130.5 (s, C-3γ), 130.2 (d, C-3δ), 113.7 (d, C-3ε), 158.2 (s, C-3ζ), 167.9 (s, 3-CO), 39.5 (q, 3-NCH_3), 55.5

(q, 3-OCH_3), 46.5 (d, C-4α), 18.4 (q, C-4β), 171.0 (s, 4-CO), 53.1 (d, C-5α), 33.1 (t, C-5β), 135.4 (s, C-5γ), 132.7 (d, C-5δa), 130.5 (d, C-5δb), 123.9 (d, C-5εa), 125.9 (d, C-5εb), 157.7 (s, C-5ζ), 169.6 (s, 5-CO), 30.1 (q, 5-NCH_3), 57.3 (d, C-6α), 35.5 (t, C-6β), 127.9 (s, C-6γ), 121.3 (d, C-6δa), 114.1 (d, C-6δb), 116.4 (d, C-6εa), 151.3 (s, C-6εb), 143.5 (s, C-6ζ), 170.5 (s, 6-CO), 29.1 (q, 6-NCH_3), 39.4 (t, C-1'), 38.5 (t, C-2'), 171.0 (s, C-3'), 35.2 (t, C-4'), 28.3 (t, C-5'), 28.1 (t, C-6'), 25.2 (t, C-7'), 55.5 (d, C-8'), 61.1 (d, C-9'), 162.8 (s, C-10'), 59.2 (d, C-11'), 39.5 (t, C-12'); ESIMS (positive): *m/z* 1105 $[M+Na]^+$; HRESIMS: *m/z* 1105.4783 (calcd for $C_{54}H_{70}N_{10}NaO_{12}S$, 1105.4793).

Biotinylated N-Boc-ethylenediamine: white amorphous powder; $C_{17}H_{30}N_4O_4S$; ^1H NMR (DMSO-d_6, 500 MHz): δ 1.29 (9H, s, H-1), 2.93 (2H, m, H-4), 3.02 (2H, m, H-5), 2.03 (2H, t, J = 7.5 Hz, H-7), 1.47 (2H, m, H-8), 1.28 (2H, m, H-9), 1.60 (2H, m, H-10), 3.08 (1H, m, H-11), 4.11 (1H, m, H-12), 4.28 (1H, m, H-14), 2.81 (1H, dd, J = 12.5, 5.0 Hz, H-15a), 2.56 (1H, d, J = 12.5 Hz, H-15b), 7.78 (1H, d, J = 5.0 Hz, 4-NH), 6.78 (1H, d, J = 5.0 Hz, 5-NH), 6.36 (1H, s, 12-NH), 6.43 (1H, s, 14-NH); ^{13}C NMR (DMSO-d_6, 125 MHz): δ 28.2 (q, C-1), 77.6 (s, C-2), 155.6 (s, C-3), 39.4 (t, C-4), 38.6 (t, C-5), 172.1 (s, C-6), 35.2 (t, C-7), 28.1 (t, C-8), 28.1 (t, C-9), 25.1 (t, C-10), 55.3 (d, C-11), 61.0 (d, C-12), 162.7 (s, C-13), 59.2 (d, C-14), 39.7 (t, C-15); ESIMS (positive): *m/z* 409 $[M+Na]^+$.

CB6: The mixture of RA-V (100 mg, 0.132 mmol), KI (catalyst), and K_2CO_3 (20.0 mg, 0.145 mmol) were dissolved in DMF (5.0 mL), following N-Boc-2-bromethanamine (50.0 mg, 0.223 mmol) was added and stirred at 50 ^oC for 12 h. The reaction solution was then purified by HPLC (45 % ACN-55 % H_2O) to yield compound **CB6** as white amorphous powder (40.0 mg, 36 %).

CB6: white amorphous powder; $C_{47}H_{61}N_7O_{11}$; ^{1}H NMR ($CDCl_3$, 400 MHz): *δ* 4.32 (1H, m, H-1α), 1.30 (3H, d, *J* = 6.5 Hz, H-1β), 6.73 (1H, d, *J* = 7.5 Hz, 1-NH), 4.74 (1H, m, H-2α), 1.35 (3H, d, *J* = 6.5 Hz, H-2β), 3.67 (1H, m, H-3α), 3.36 (2H, m, H-3β), 7.03 (2H, d, *J* = 8.0 Hz, H-3δ), 6.82 (2H, d, *J* = 8.0 Hz, H-3ε), 2.85 (3H, s, 3-NCH_3), 3.78 (3H, s, 3-OCH_3), 4.75 (1H, t, *J* = 6.5 Hz, H-4α), 1.08 (3H, d, *J* = 6.5 Hz, H-4β), 6.47 (d, *J* = 6.5 Hz, 4-NH), 5.40 (1H, dd, *J* = 11.0, 2.0 Hz, H-5α), 2.66 (1H, overlap, H-5βa), 3.68 (1H, overlap, H-5βb), 7.25 (1H, overlap, H-5δa), 7.40 (1H, dd, *J* = 8.0, 1.5 Hz, H-5δb), 6.83 (1H, overlap, H-5εa), 7.21 (1H, dd, *J* = 8.0, 2.0 Hz, H-5εb), 3.11 (3H, s, 5-NCH_3), 4.55 (1H, dd, *J* = 11.5, 3.0 Hz, H-6α), 3.08 (1H, overlap, H-6βa), 2.96 (1H, overlap, H-6βb), 6.55 (1H, d, *J* = 7.0 Hz, H-6δa), 4.32 (1H, br s, H-6δb), 6.83 (1H, overlap, H-6εa), 2.65 (3H, s, 6-NCH_3), 4.13 (2H, t, *J* = 4.5 Hz, H-1'), 3.59 (2H, m, H-2'), 1.44 (9H, s, H-5'); ^{13}C NMR ($CDCl_3$, 100 MHz): *δ* 47.7 (d, C-1α), 20.7 (q, C-1β), 172.2 (s, 1-CO), 44.5 (d, C-2α), 16.5 (q, C-2β), 172.7 (s, 2-CO), 68.3 (d, C-3α), 32.5 (t, C-3β), 130.5 (s, C-3γ), 130.2 (d, C-3δ), 114.0 (d, C-3ε), 158.3 (s, C-3ζ), 168.1 (s, 3-CO), 39.8 (q, 3-NCH_3), 55.2 (q, 3-OCH_3), 46.4 (d, C-4α), 18.5 (q, C-4β), 171.6 (s, 4-CO), 54.2 (d, C-5α), 36.9 (t, C-5β), 135.2 (s, C-5γ), 132.8 (d, C-5δa), 131.0 (d, C-5δb), 124.2 (d, C-5εa), 125.9 (d, C-5εb), 158.0 (s, C-5ζ), 169.3 (s, 5-CO), 30.5 (q, 5-NCH_3), 57.2 (d, C-6α), 35.5 (t, C-6β), 129.0 (s, C-6γ), 121.1 (d, C-6δa), 113.6 (d, C-6δb), 115.8 (d, C-6εa), 153.6 (s, C-6εb), 145.4 (s, C-6ζ), 170.6 (s, 6-CO), 29.3 (q, 6-NCH_3), 69.2 (t, C-1'), 40.2 (t, C-2'), 156.0 (s, C-3'), 79.5 (s, C-4'), 28.4 (q, C-5'); ESIMS (positive): *m/z* 922 $[M+Na]^+$; HRESIMS: *m/z* 922.4325 (calcd for $C_{47}H_{61}N_7NaO_{11}$, 922.4327).

CB7: The mixture of RA-V (20.0 mg, 0.026 mmol), KI (catalyst), and NaOH

(5.0 mg, 0.125 mmol) were dissolved in DMF (1.0 mL), following tert-butyl bromoacetate (7.0 μL, 0.043 mmol) was added and stirred at 45 ^{o}C for 12 h. The reaction solution was then purified by HPLC (70 % ACN-30 % H_2O) to yield compound **CB7** as white amorphous powder (12.0 mg, 52 %).

CB7: white amorphous powder; $C_{46}H_{58}N_6O_{11}$; ^{1}H NMR ($CDCl_3$, 400 MHz): δ 4.38 (1H, m, H-1α), 1.27 (3H, d, J = 6.5 Hz, H-1β), 6.72 (1H, overlap, 1-NH), 4.79 (1H, m, H-2α), 1.33 (3H, d, J = 6.5 Hz, H-2β), 3.57 (1H, dd, J = 10.5, 5.0 Hz, H-3α), 3.33 (2H, m, H-3β), 7.02 (2H, d, J = 8.5 Hz, H-3δ), 6.81 (2H, d, J = 8.5 Hz, H-3ε), 2.84 (3H, s, 3-NCH_3), 3.77 (3H, s, 3-OCH_3), 4.75 (1H, m, H-4α), 1.07 (3H, d, J = 6.5 Hz, H-4β), 6.48 (d, J = 7.0 Hz, 4-NH), 5.39 (1H, dd, J = 11.0, 2.5 Hz, H-5α), 2.64 (1H, overlap, H-5βa), 3.65 (1H, t, J = 11.0 Hz, H-5βb), 7.24 (1H, dd, J = 8.5, 2.0 Hz, H-5δa), 7.40 (1H, dd, J = 8.5, 2.0 Hz, H-5δb), 6.81 (1H, overlap, H-5εa), 7.19 (1H, dd, J = 8.5, 2.0 Hz, H-5εb), 3.10 (3H, s, 5-NCH_3), 4.53 (1H, dd, J = 12.0, 3.5 Hz, H-6α), 3.08 (1H, overlap, H-6βa), 2.93 (1H, overlap, H-6βb), 6.52 (1H, d, J = 8.5 Hz, H-6δa), 4.33 (1H, br s, H-6δb), 6.86 (1H, dd, J = 8.5, 2.0 Hz, H-6εa), 2.66 (3H, s, 6-NCH_3), 2.67 (2H, overlap, H-1'), 1.46 (9H, s, H-4'); ^{13}C NMR ($CDCl_3$, 100 MHz): δ 47.7 (d, C-1α), 20.7 (q, C-1β), 172.2 (s, 1-CO), 44.5 (d, C-2α), 16.4 (q, C-2β), 172.6 (s, 2-CO), 68.2 (d, C-3α), 32.5 (t, C-3β), 130.5 (s, C-3γ), 130.2 (d, C-3δ), 114.0 (d, C-3ε), 158.3 (s, C-3ζ), 168.1 (s, 3-CO), 39.8 (q, 3-NCH_3), 55.2 (q, 3-OCH_3), 46.3 (d, C-4α), 18.4 (q, C-4β), 171.7 (s, 4-CO), 54.2 (d, C-5α), 36.9 (t, C-5β), 135.1 (s, C-5γ), 132.7 (d, C-5δa), 131.0 (d, C-5δb), 124.2 (d, C-5εa), 125.9 (d, C-5εb), 158.1 (s, C-5ζ), 169.3 (s, 5-CO), 30.5 (q, 5-NCH_3), 57.2 (d, C-6α), 35.5 (t, C-6β), 129.4 (s, C-6γ), 120.8 (d, C-6δa), 113.8 (d, C-6δb), 114.8 (d, C-6εa), 153.4 (s, C-6εb), 144.8 (s, C-6ζ), 170.6 (s, 6-CO), 29.3 (q, 6-NCH_3), 66.9 (t, C-1'), 168.0 (s, C-2'),

82.3 (s, C-3'), 28.0 (s, C-4'); ESIMS (positive): *m/z* 893 $[M+Na]^+$; HRESIMS: *m/z* 893.4051 (calcd for $C_{46}H_{58}N_6NaO_{11}$, 893.4061).

CB8: The mixture of RA-V (10.0 mg, 0.013 mmol), KI (catalyst), and K_2CO_3 (5.0 mg, 0.036 mmol) were dissolved in DMF (1.0 mL), following propargyl bromide (10.0 *μ*L, 0.127 mmol) was added and stirred at 60 ^oC for 12 h. The reaction solution was then purified by HPLC (60 % ACN-40 % H_2O) to yield compound **CB8** as white amorphous powder (6.3 mg, 60 %).

CB8: white amorphous powder; $C_{43}H_{50}N_6O_9$; ^1H NMR ($CDCl_3$, 600 MHz): *δ* 4.33 (1H, t, *J* = 7.0 Hz, H-1α), 1.28 (3H, d, *J* = 7.0 Hz, H-1β), 6.69 (1H, d, *J* = 7.0 Hz, 1-NH), 4.83 (1H, m, H-2α), 1.33 (3H, d, *J* = 7.0 Hz, H-2β), 6.43 (1H, d, *J* = 7.0 Hz, 2-NH), 3.56 (1H, dd, *J* = 10.5, 4.5 Hz, H-3α), 3.35 (2H, m, H-3β), 7.02 (2H, d, *J* = 8.0 Hz, H-3δ), 6.82 (2H, d, *J* = 8.0 Hz, H-3ε), 2.83 (3H, s, 3-NCH_3), 3.77 (3H, s, 3-OCH_3), 4.72 (1H, t, *J* = 7.0 Hz, H-4α), 1.09 (3H, d, *J* = 6.5 Hz, H-4β), 6.34 (d, *J* = 8.5 Hz, 4-NH), 5.39 (1H, dd, *J* = 11.5, 2.5 Hz, H-5α), 2.62 (1H, dd, *J* = 11.5, 2.5 Hz, H-5βa), 3.65 (1H, overlap, H-5βb), 7.25 (1H, dd, *J* = 8.0, 2.0 Hz, H-5δa), 7.40 (1H, dd, *J* = 8.5, 2.0 Hz, H-5δb), 6.82 (1H, overlap, H-5εa), 7.18 (1H, dd, *J* = 8.5, 2.5 Hz, H-5εb), 3.10 (3H, s, 5-NCH_3), 4.53 (1H, dd, *J* = 11.0, 4.0 Hz, H-6α), 3.07 (1H, overlap, H-6βa), 2.95 (1H, overlap, H-6βb), 6.56 (1H, dd, *J* = 8.5, 2.0 Hz, H-6δa), 4.32 (1H, br s, H-6δb), 6.85 (1H, dd, *J* = 8.5, 2.0 Hz, H-6εa), 2.67 (3H, s, 6-NCH_3), 4.81 (2H, overlap, H-1'), 2.54 (1H, t, *J* = 2.5 Hz, H-3'); ^{13}C NMR ($CDCl_3$, 150 MHz): *δ* 48.0 (d, C-1α), 20.9 (q, C-1β), 172.4 (s, 1-CO), 44.7 (d, C-2α), 16.8 (q, C-2β), 172.8 (s, 2-CO), 68.5 (d, C-3α), 32.9 (t, C-3β), 130.8 (s, C-3γ), 130.4 (d, C-3δ), 114.2 (d, C-3ε), 158.6 (s, C-3ζ), 168.2 (s, 3-CO), 40.0 (q, 3-NCH_3), 55.5 (q, 3-OCH_3), 46.6 (d, C-4α), 18.8 (q, C-4β), 171.9 (s, 4-CO), 54.4 (d, C-5α), 37.2 (t, C-5β), 135.4 (s, C-5γ),

133.0 (d, C-5δa), 131.2 (d, C-5δb), 124.4 (d, C-5εa), 126.1 (d, C-5εb), 158.3 (s, C-5ζ), 169.5 (s, 5-CO), 30.8 (q, 5-NCH_3), 57.4 (d, C-6α), 35.7 (t, C-6β), 129.8 (s, C-6γ), 121.0 (d, C-6δa), 113.8 (d, C-6δb), 115.5 (d, C-6εa), 153.9 (s, C-6εb), 144.4 (s, C-6ζ), 170.8 (s, 6-CO), 29.5 (q, 6-NCH_3), 57.4 (t, C-1'), 78.7 (s, C-2'), 76.3 (d, C-3'); ESIMS (positive): *m/z* 817 $[M+Na]^+$; HRESIMS: *m/z* 817.3529 (calcd for $C_{43}H_{50}N_6NaO_9$, 817.3537).

CB9: The mixture of RA-V (10.0 mg, 0.013 mmol), KI (catalyst), and KOH (3.0 mg, 0.054 mmol) were dissolved in DMF (1.0 mL), following 2-[(2-chloroethoxy)ethoxyl]ethanol (3.0 μL, 0.021 mmol) was added and stirred at 40 ^oC for 12 h. The reaction solution was then purified by HPLC (40 % ACN-60 % H_2O) to yield compound **CB9** as white amorphous powders (6.2 mg, 53 %).

CB9: white amorphous powder; $C_{46}H_{60}N_6O_{12}$; ^1H NMR ($CDCl_3$, 600 MHz): *δ* 4.83 (1H, m, H-1α), 1.28 (3H, d, *J* = 7.0 Hz, H-1β), 6.70 (1H, d, *J* = 7.5 Hz, 1-NH), 4.34 (1H, t, *J* = 7.0 Hz, H-2α), 1.33 (3H, d, *J* = 6.5 Hz, H-2β), 6.43 (1H, d, *J* = 6.5 Hz, 2-NH), 3.57 (1H, dd, *J* = 11.0, 4.5 Hz, H-3α), 3.35 (2H, m, H-3β), 7.02 (2H, d, *J* = 8.5 Hz, H-3δ), 6.82 (2H, d, *J* = 8.5 Hz, H-3ε), 2.84 (3H, s, 3-NCH_3), 3.77 (3H, s, 3-OCH_3), 4.72 (1H, m, H-4α), 1.08 (3H, d, *J* = 6.5 Hz, H-4β), 6.41 (d, *J* = 8.5 Hz, 4-NH), 5.38 (1H, dd, *J* = 11.5, 3.0 Hz, H-5α), 2.62 (1H, overlap, H-5βa), 3.62 (1H, overlap, H-5βb), 7.25 (1H, overlap, H-5δa), 7.39 (1H, dd, *J* = 8.0, 2.0 Hz, H-5δb), 6.82 (1H, overlap, H-5εa), 7.18 (1H, dd, *J* = 8.0, 2.0 Hz, H-5εb), 3.10 (3H, s, 5-NCH_3), 4.52 (1H, dd, *J* = 12.0, 4.0 Hz, H-6α), 3.08 (1H, overlap, H-6βa), 2.94 (1H, overlap, H-6βb), 6.53 (1H, dd, *J* = 8.5, 2.0 Hz, H-6δa), 4.31 (1H, br s, H-6δb), 6.84 (1H, dd, *J* = 8.5, 2.0 Hz, H-6εa), 2.66 (3H, s, 6-NCH_3), 4.24 (2H, t, *J* = 4.0 Hz, H-1'), 3.72 (2H, t, *J* = 4.0

Hz, H-2'), 3.93 (2H, t, J = 4.0 Hz, H-3'), 3.71 (2H, m, H-4'), 3.77 (2H, overlap, H-5'), 3.62 (2H, overlap, H-6'); ^{13}C NMR ($CDCl_3$, 150 MHz): δ 48.0 (d, C-1α), 20.9 (q, C-1β), 172.5 (s, 1-CO), 44.8 (d, C-2α), 16.8 (q, C-2β), 172.9 (s, 2-CO), 68.6 (d, C-3α), 32.8 (t, C-3β), 130.8 (s, C-3γ), 130.4 (d, C-3δ), 114.2 (d, C-3ε), 158.6 (s, C-3ζ), 168.1 (s, 3-CO), 40.1 (q, 3-NCH_3), 55.5 (q, 3-OCH_3), 46.6 (d, C-4α), 18.6 (q, C-4β), 171.9 (s, 4-CO), 54.4 (d, C-5α), 37.1 (t, C-5β), 135.3 (s, C-5γ), 133.0 (d, C-5δa), 131.2 (d, C-5δb), 124.5 (d, C-5εa), 126.2 (d, C-5εb), 158.4 (s, C-5ζ), 169.5 (s, 5-CO), 30.6 (q, 5-NCH_3), 57.5 (d, C-6α), 35.7 (t, C-6β), 129.5 (s, C-6γ), 121.2 (d, C-6δa), 113.8 (d, C-6δb), 115.0 (d, C-6εa), 153.8 (s, C-6εb), 145.8 (s, C-6ζ), 170.8 (s, 6-CO), 29.5 (q, 6-NCH_3), 72.7 (t, C-1'), 69.1 (t, C-2'), 71.1 (t, C-3'), 70.6 (t, C-4'), 70.0 (t, C-5'), 62.0 (t, C-6'); ESIMS (positive): m/z 911 $[M+Na]^+$.

CB10: The mixture of RA-V (10.0 mg, 0.013 mmol), KI (catalyst), and KOH (3.0 mg, 0.054 mmol) were dissolved in DMF (1.0 mL), following propargyl bromide (10.0 μL, 0.127 mmol) was added and stirred at 50 ^{o}C for 12 h. The reaction solution was then purified by HPLC (40 % ACN-60 % H_2O) to yield compound **CB10** as white amorphous powder (6.4 mg, 60 %).

CB10: white amorphous powder; $C_{46}H_{52}N_6O_9$; ^{1}H NMR ($CDCl_3$, 600 MHz): δ 4.79 (1H, overlap, H-1α), 1.40 (3H, d, J = 6.5 Hz, H-1β), 6.64 (1H, d, J = 7.5 Hz, 1-NH), 5.08 (1H, J = 7.0 Hz, H-2α), 1.56 (3H, d, J = 7.0 Hz, H-2β), 3.53 (1H, dd, J = 11.0, 4.5 Hz, H-3α), 3.31 (2H, m, H-3β), 7.03 (2H, d, J = 8.5 Hz, H-3δ), 6.82 (2H, d, J = 8.5 Hz, H-3ε), 2.81 (3H, s, 3-NCH_3), 3.78 (3H, s, 3-OCH_3), 4.79 (1H,overlap, H-4α), 0.92 (3H, d, J = 6.5 Hz, H-4β), 5.42 (1H, dd, J = 10.5, 2.5 Hz, H-5α), 2.62 (1H, dd, J = 11.0, 2.5 Hz, H-5βa), 3.70 (1H, m, H-5βb), 7.25 (1H, dd, J = 8.0, 2.0 Hz, H-5δa), 7.39 (1H, dd, J = 8.0, 2.5 Hz, H-5δb),

6.83 (1H, overlap, H-5εa), 7.18 (1H, dd, J = 8.0, 2.5 Hz, H-5εb), 3.06 (3H, s, 5-NCH_3), 4.54 (1H, dd, J = 12.0, 5.0 Hz, H-6α), 3.13 (1H, overlap, H-6βa), 2.88 (1H, overlap, H-6βb), 6.56 (1H, dd, J = 8.5, 2.0 Hz, H-6δa), 4.32 (1H, br s, H-6δb), 6.82 (1H, overlap, H-6εa), 2.75 (3H, s, 6-NCH_3), 4.82 (2H, d, J = 2.5 Hz, H-1'), 2.54 (1H, t, J = 2.5 Hz, H-3'), 4.76 (1H, overlap, H-1''a), 4.08 (1H, dd, J = 19.5, 2.5 Hz, H-1''b), 2.32 (1H, t, J = 2.5 Hz, H-3''); ^{13}C NMR ($CDCl_3$, 150 MHz): δ 46.3 (d, C-1α), 20.0 (q, C-1β), 173.4 (s, 1-CO), 49.6 (d, C-2α), 14.8 (q, C-2β), 172.9 (s, 2-CO), 68.2 (d, C-3α), 32.8 (t, C-3β), 130.9 (s, C-3γ), 130.5 (d, C-3δ), 114.2 (d, C-3ε), 158.6 (s, C-3ζ), 168.6 (s, 3-CO), 39.5 (q, 3-NCH_3), 55.5 (q, 3-OCH_3), 46.4 (d, C-4α), 18.6 (q, C-4β), 171.9 (s, 4-CO), 54.8 (d, C-5α), 37.0 (t, C-5β), 135.8 (s, C-5γ), 133.0 (d, C-5δa), 131.1 (d, C-5δb), 124.3 (d, C-5εa), 126.1 (d, C-5εb), 158.2 (s, C-5ζ), 169.0 (s, 5-CO), 30.8 (q, 5-NCH_3), 57.4 (d, C-6α), 36.0 (t, C-6β), 129.8 (s, C-6γ), 121.2 (d, C-6δa), 113.8 (d, C-6δb), 115.5 (d, C-6εa), 153.9 (s, C-6εb), 144.5 (s, C-6ζ), 170.9 (s, 6-CO), 29.8 (q, 6-NCH_3), 57.4 (t, C-1'), 78.7 (s, C-2'), 76.3 (d, C-3'), 34.5 (t, C-1''), 80.3 (s, C-2''), 73.5 (d, C-3''); ESIMS (positive): *m/z* 855 $[M+Na]^+$; HRESIMS: *m/z* 855.3685 (calcd for $C_{46}H_{52}N_6NaO_9$, 855.3694).

CB11: The mixture of RA-VII (10.0 mg, 0.013 mmol), KI (catalyst), and KOH (3.0 mg, 0.054 mmol) were dissolved in DMF (1.0 mL), following propargyl bromide (10.0 μL, 0.127 mmol) was added and stirred at 50 ^oC for 12 h. The reaction solution was then purified by HPLC (45 % ACN-55 % H_2O) to yield compound **CB11** as white amorphous powder (6.4 mg, 60 %).

CB11: white amorphous powder; $C_{44}H_{52}N_6O_9$; ^1H NMR ($CDCl_3$, 600 MHz): δ 4.79 (1H, overlap, H-1α), 1.40 (3H, d, J = 7.0 Hz, H-1β), 6.64 (1H, d, J = 7.5 Hz, 1-NH), 5.09 (1H, J = 7.5 Hz, H-2α), 1.56 (3H, d, J = 7.5 Hz, H-2β), 3.53 (1H,

dd, *J* = 11.0, 5.0 Hz, H-3α), 3.31 (2H, m, H-3β), 7.03 (2H, d, *J* = 8.5 Hz, H-3δ), 6.82 (2H, d, *J* = 8.5 Hz, H-3ε), 2.81 (3H, s, 3-NCH_3), 3.78 (3H, s, 3-OCH_3), 4.79 (1H,overlap, H-4α), 0.92 (3H, d, *J* = 6.5 Hz, H-4β), 5.43 (1H, dd, *J* = 11.0, 2.5 Hz, H-5α), 2.61 (1H, dd, *J* = 11.0, 2.5 Hz, H-5βa), 3.70 (1H, m, H-5βb), 7.25 (1H, dd, *J* = 8.5, 2.0 Hz, H-5δa), 7.39 (1H, dd, *J* = 8.5, 2.0 Hz, H-5δb), 6.85 (1H, dd, *J* = 8.5, 2.0 Hz, H-5εa), 7.19 (1H, dd, *J* = 8.5, 2.5 Hz, H-5εb), 3.06 (3H, s, 5-NCH_3), 4.55 (1H, dd, *J* = 12.0, 4.0 Hz, H-6α), 3.13 (1H, overlap, H-6βa), 2.89 (1H, overlap, H-6βb), 6.56 (1H, dd, *J* = 8.5, 2.0 Hz, H-6δa), 4.30 (1H, d, *J* = 2.0 Hz, H-6δb), 6.78 (1H, d, *J* = 8.5 Hz, H-6εa), 2.73 (3H, s, 6-NCH_3), 3.92 (3H, s, 6-OCH_3), 4.76 (1H, overlap, H-1'a), 4.09 (1H, dd, *J* = 19.0, 2.5 Hz, H-1'b), 2.32 (1H, t, *J* = 2.5 Hz, H-3'); ^{13}C NMR ($CDCl_3$, 150 MHz): *δ* 46.3 (d, C-1α), 20.0 (q, C-1β), 173.4 (s, 1-CO), 49.5 (d, C-2α), 14.8 (q, C-2β), 172.9 (s, 2-CO), 68.2 (d, C-3α), 32.8 (t, C-3β), 130.9 (s, C-3γ), 130.5 (d, C-3δ), 114.2 (d, C-3ε), 158.6 (s, C-3ζ), 168.5 (s, 3-CO), 39.5 (q, 3-NCH_3), 55.5 (q, 3-OCH_3), 46.3 (d, C-4α), 18.6 (q, C-4β), 171.9 (s, 4-CO), 54.8 (d, C-5α), 37.0 (t, C-5β), 135.7 (s, C-5γ), 133.1 (d, C-5δa), 131.1 (d, C-5δb), 124.3 (d, C-5εa), 126.1 (d, C-5εb), 158.3 (s, C-5ζ), 169.0 (s, 5-CO), 30.8 (q, 5-NCH_3), 57.3 (d, C-6α), 35.9 (t, C-6β), 128.3 (s, C-6γ), 121.3 (d, C-6δa), 112.3 (d, C-6δb), 113.4 (d, C-6εa), 153.2 (s, C-6εb), 146.7 (s, C-6ζ), 170.9 (s, 6-CO), 29.8 (q, 6-NCH_3), 56.3 (q, 6-OCH_3), 34.5 (t, C-1'), 80.3 (s, C-2'), 73.5 (d, C-3'); ESIMS (positive): *m/z* 831 $[M+Na]^+$; HRESIMS: *m/z* 831.3686 (calcd for $C_{44}H_{52}N_6NaO_9$, 831.3694).

CB12: Compound **CB6** (20.0 mg, 0.022 mmol) was dissolved in TFA-CH_2Cl_2 (30 %, 2 mL) and stirred at room temperature for 2 h. After the solution was evaporated, biotin-NHS (10.0 mg, 0.029 mmol) was added and

dissolved in ACN (3 mL), and then stirred at room temperature for 12 h. The reaction solution was then purified by HPLC (30 % ACN-70 % H_2O) to yield compound **CB12** as white amorphous powder (20.0 mg, 92 %).

CB12: white amorphous powder; $C_{52}H_{67}N_9O_{11}S$; ^{1}H NMR (C_5D_5N, 600 MHz): δ 5.15 (1H, overlap, H-1α), 1.58 (3H, d, *J* = 6.5 Hz, H-1β), 8.78 (1H, d, *J* = 8.0 Hz, 1-NH), 5.14 (1H, overlap, H-2α), 1.47 (3H, d, *J* = 7.0 Hz, H-2β), 9.97 (1H, d, *J* = 8.5 Hz, 2-NH), 4.11 (1H, dd, *J* = 11.0, 4.5 Hz, H-3α), 3.93 (1H, overlap, H-3βa), 3.81 (1H, dd, *J* = 14.0, 4.5 Hz, H-3βb), 7.28 (2H, d, *J* = 8.5 Hz, H-3δ), 7.02 (2H, d, *J* = 8.5 Hz, H-3ε), 3.17 (3H, s, 3-NCH_3), 3.71 (3H, s, 3-OCH_3), 5.14 (1H, overlap, H-4α), 1.38 (3H, d, *J* = 6.5 Hz, H-4β), 7.40 (1H, d, *J* = 8.0 Hz, 4-NH), 5.77 (1H, dd, *J* = 11.5, 2.5 Hz, H-5α), 3.66 (1H, t, *J* = 11.5 Hz, H-5βa), 2.62 (1H, dd, *J* = 11.5, 2.5 Hz, H-5βb), 7.49 (1H, dd, *J* = 8.5, 2.0 Hz, H-5δa), 7.31 (1H, dd, *J* = 8.0, 2.0 Hz, H-5δb), 6.97 (1H, dd, *J* = 8.5, 2.5 Hz, H-5εa), 7.02 (1H, d, *J* = 8.0 Hz, H-5εb), 3.01 (3H, s, 5-NCH_3), 5.03 (1H, dd, *J* = 12.0, 3.5 Hz, H-6α), 3.56 (1H, dd, *J* = 17.0, 3.5 Hz, H-6βa), 3.36 (1H, dd, *J* = 17.0, 12.0 Hz, H-6βb), 6.76 (1H, dd, *J* = 8.5, 1.5 Hz, H-6δa), 4.61 (1H, br s, H-6δb), 6.89 (1H, d, *J* = 8.5 Hz, H-6εa), 3.02 (3H, s, 6-NCH_3), 4.37 (2H, t, *J* = 4.5 Hz, H-1'), 3.96 (2H, m, H-2'), 9.20 (1H, t, *J* = 6.0 Hz, 2'-NH), 2.40 (2H, t, *J* = 6.0 Hz, H-4'), 1.81 (2H, m, H-5'), 1.59 (2H, m, H-6'), 1.89 (1H, m, H-7'a), 1.81 (1H, m, H-7'b), 3.19 (1H, m, H-8'), 4.35 (1H, m, H-9'), 4.52 (1H, m, H-11'), 2.92 (2H, m, H-12'), 7.61 (1H, br s, 9'-NH), 7.52 (1H, br s, 11'-NH); ^{13}C NMR (C_5D_5N, 150 MHz): δ 48.6 (d, C-1α), 22.2 (q, C-1β), 173.1 (s, 1-CO), 45.1 (d, C-2α), 17.4 (q, C-2β), 173.9 (s, 2-CO), 69.0 (d, C-3α), 34.0 (t, C-3β), 132.3 (s, C-3γ), 131.3 (d, C-3δ), 114.9 (d, C-3ε), 159.3 (s, C-3ζ), 169.4 (s, 3-CO), 40.4 (q, 3-NCH_3), 55.6 (q, 3-OCH_3), 47.4 (d, C-4α), 19.5 (q, C-4β), 172.5 (s, 4-CO),

55.0 (d, C-5α), 37.2 (t, C-5β), 136.7 (s, C-5γ), 134.0 (d, C-5δa), 131.7 (d, C-5δb), 125.0 (d, C-5εa), 127.0 (d, C-5εb), 159.0 (s, C-5ζ), 170.4 (s, 5-CO), 30.9 (q, 5-NCH_3), 58.2 (d, C-6α), 36.6 (t, C-6β), 130.8 (s, C-6γ), 122.1 (d, C-6δa), 115.2 (d, C-6δb), 115.0 (d, C-6εa), 154.1 (s, C-6εb), 146.6 (s, C-6ζ), 171.7 (s, 6-CO), 30.1 (q, 6-NCH_3), 69.3 (t, C-1'), 40.1 (t, C-2'), 173.9 (s, C-3'), 36.6 (t, C-4'), 26.5 (t, C-5'), 29.4 (t, C-6'), 29.5 (t, C-7'), 56.7 (d, C-8'), 62.8 (d, C-9'), 164.9 (s, C-10'), 61.0 (d, C-11'), 41.6 (t, C-12'); ESIMS (positive): *m/z* 1048 $[M+Na]^+$; HRESIMS: *m/z* 1048.4577 (calcd for $C_{52}H_{67}N_9NaO_{11}S$, 1048.4578).

IV. Supplementary Chemical Compound Information

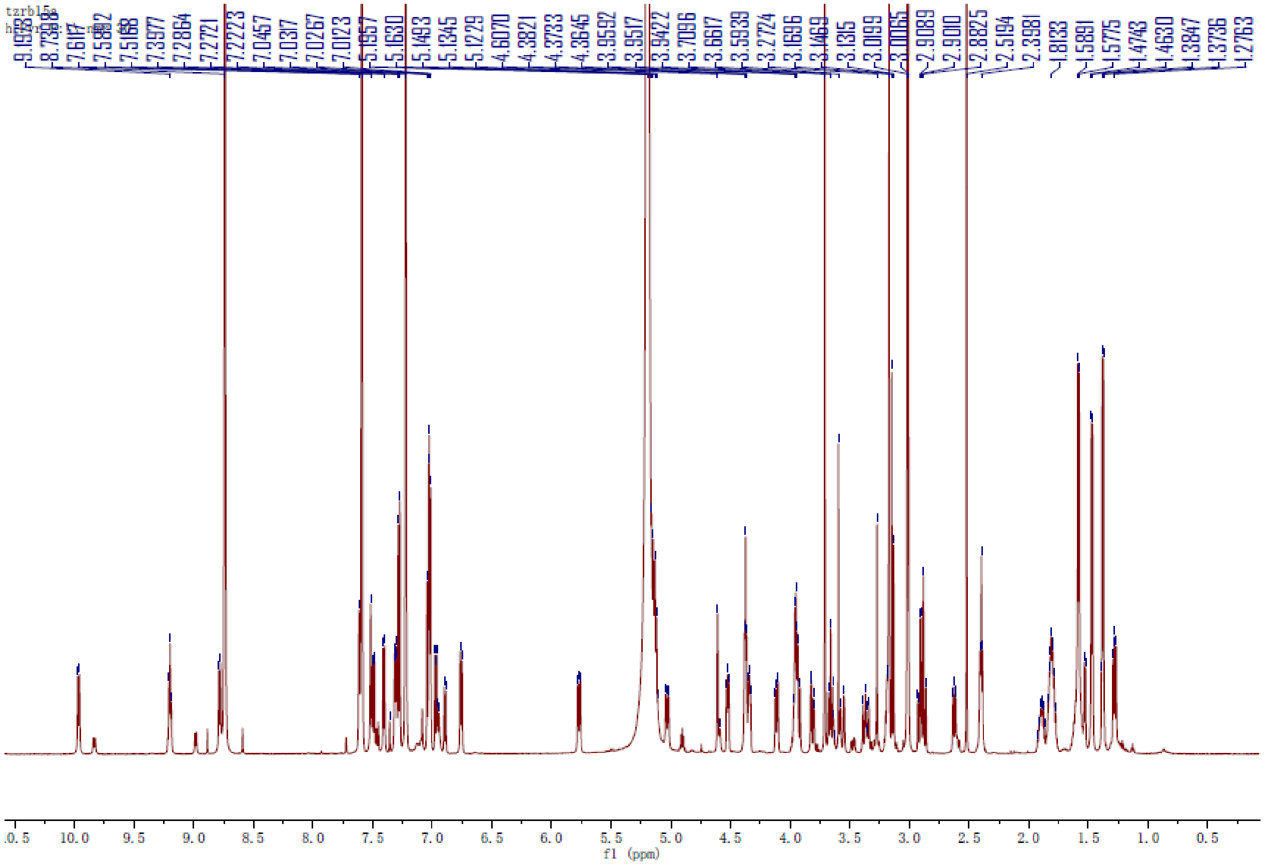

^{1}H NMR spectrum of compound **CB12**

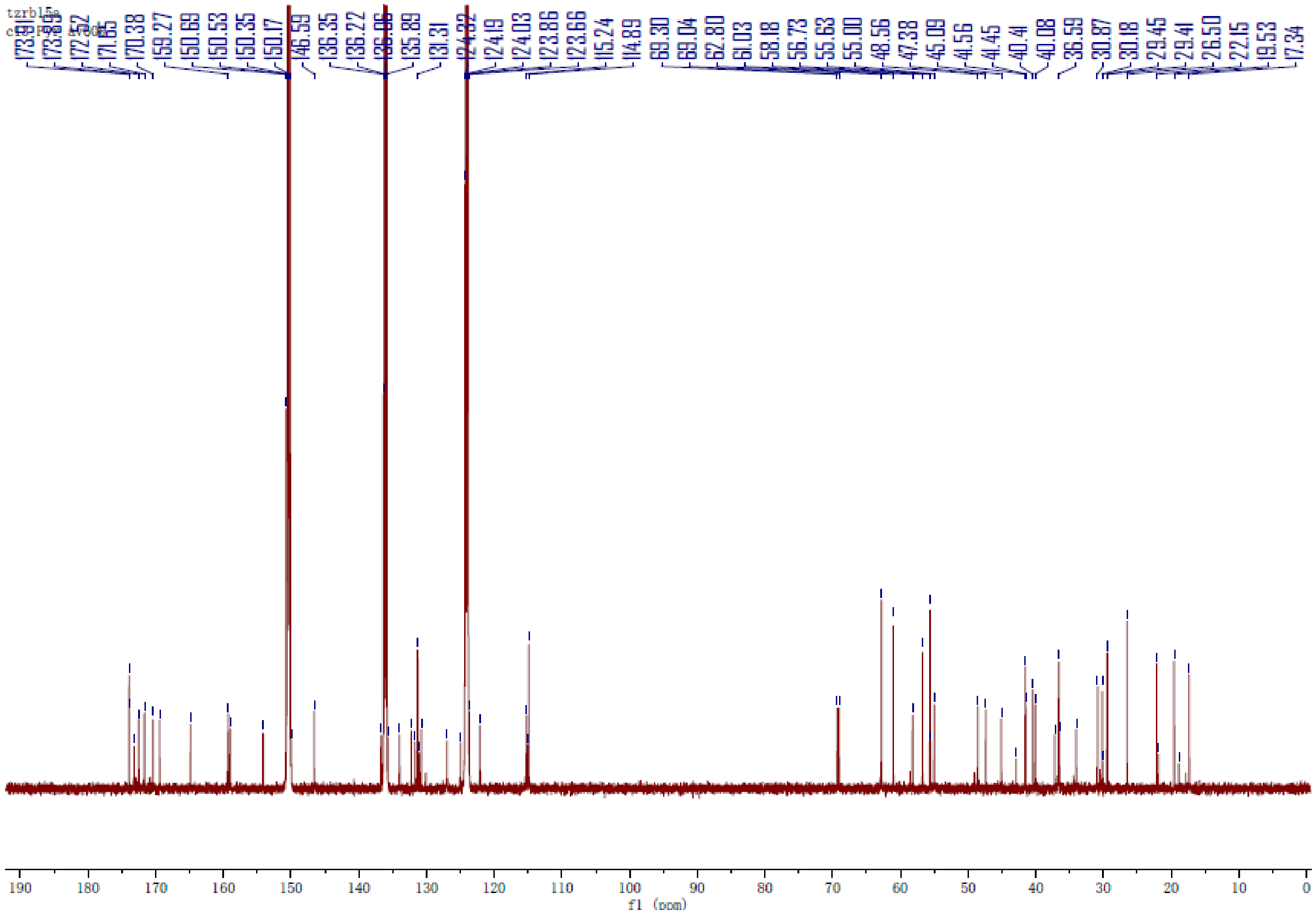

^{13}C NMR spectrum of compound **CB12**

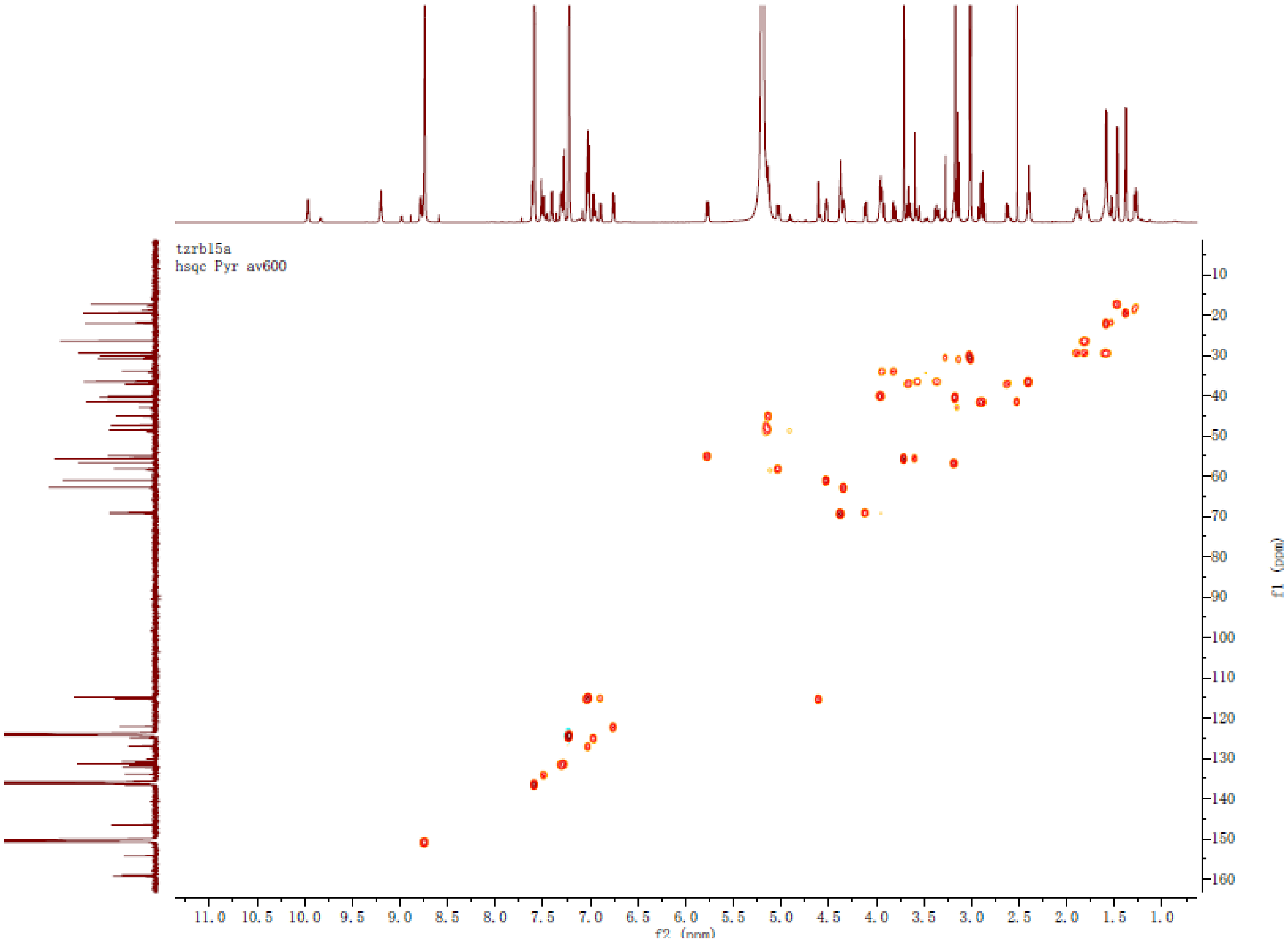

HMQC spectrum of compound **CB12**

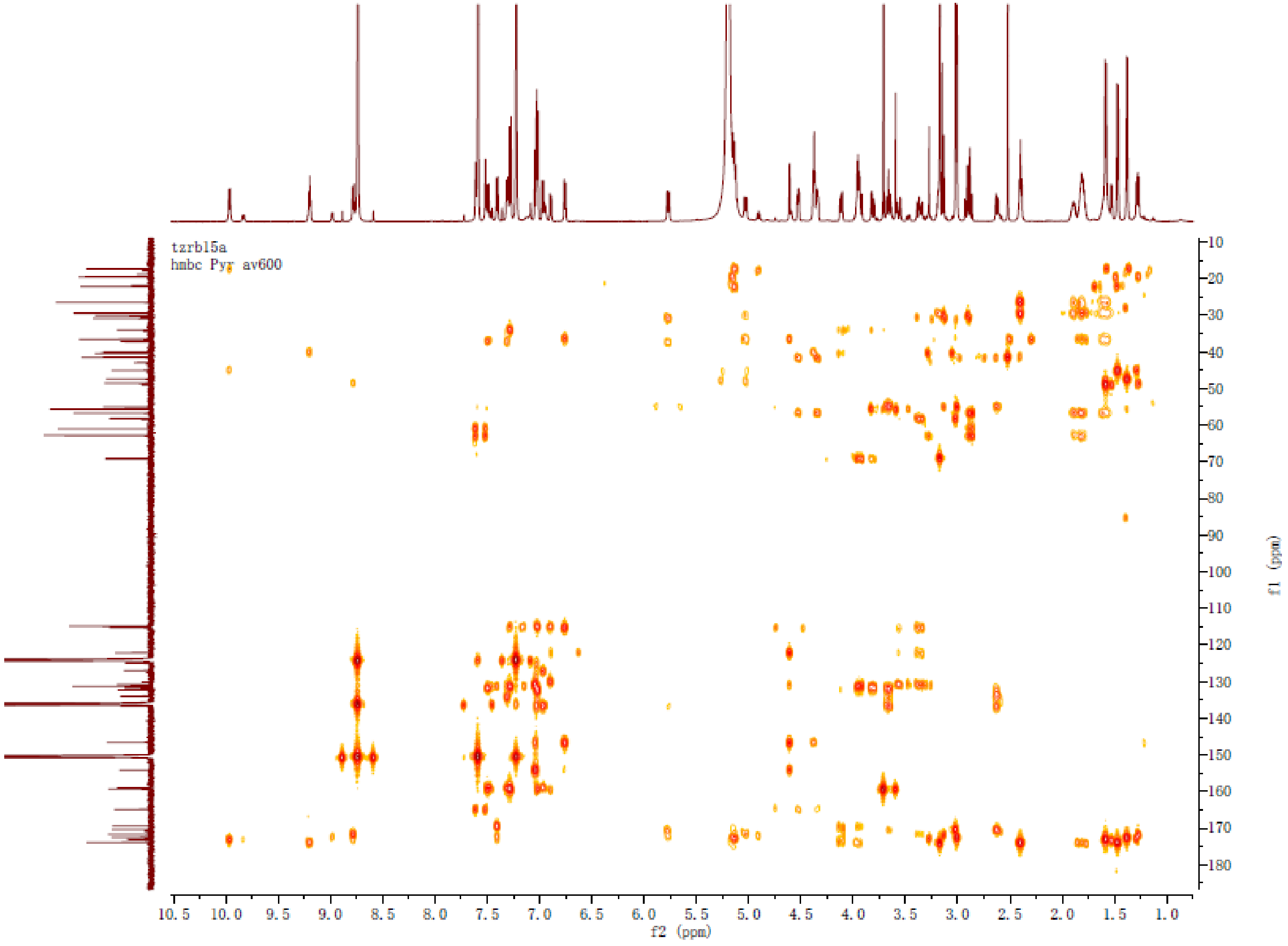

HMBC spectrum of compound **CB12**

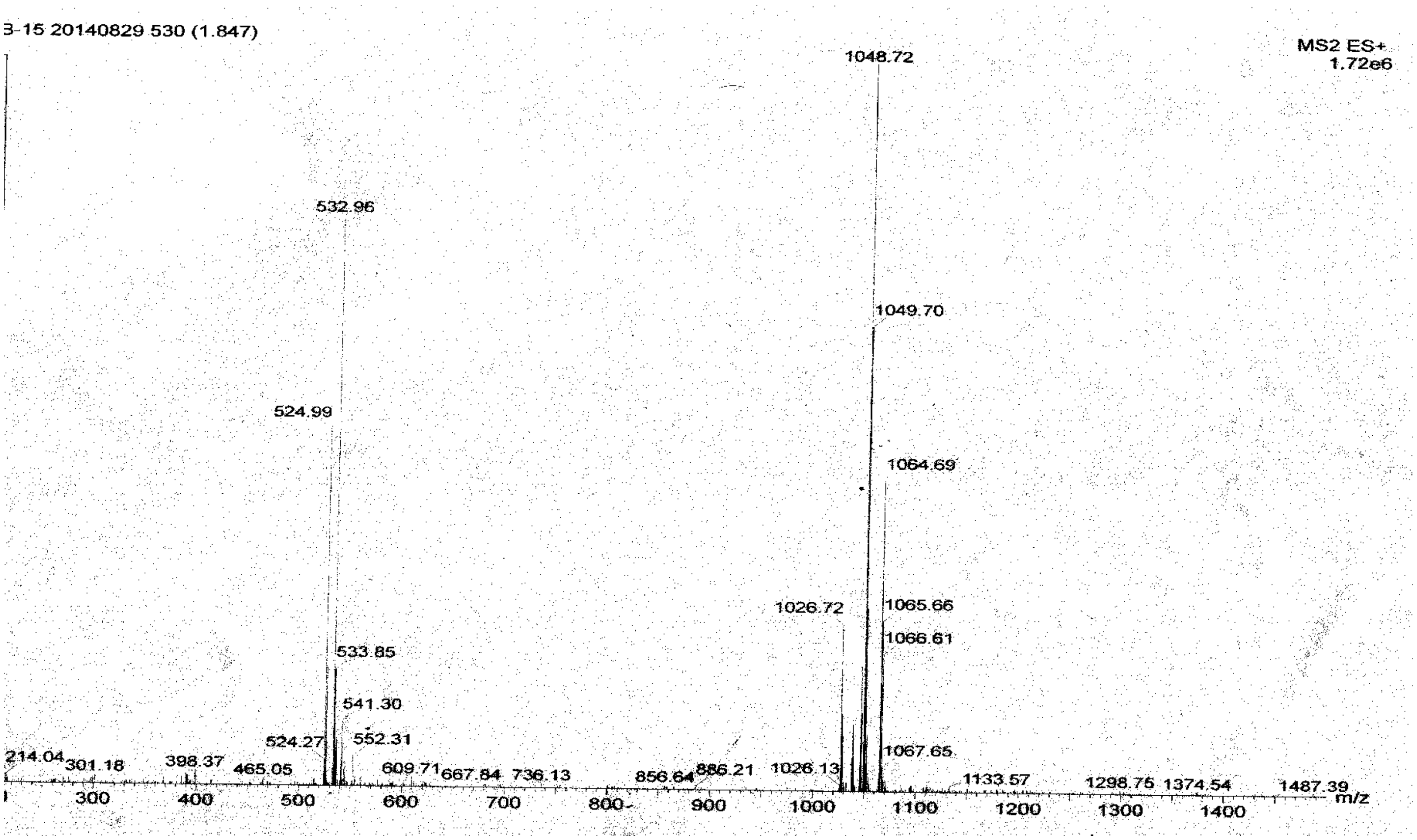

ESI-MS spectrum of **CB12**

**Data Filename**	140903ESIA7.d	**Sample Name**	TZRB-15
**Sample Type**	Sample	**Position**	
**Instrument Name**	Agilent G6230 TOF MS	**User Name**	KIB
**Acq Method**	ESI.m	**Acquired Time**	9/3/2014 12:11:11 PM
**IRM Calibration Status**	Success	**DA Method**	demo.m
**Comment**			

Sample Group **Info.**

Acquisition SW Version 6200 series TOF/6500 series Q-TOF B.05.01 (B5125.2)

User Spectra

Fragmentor Voltage	Collision Energy	Ionization Mode
200	0	ESI

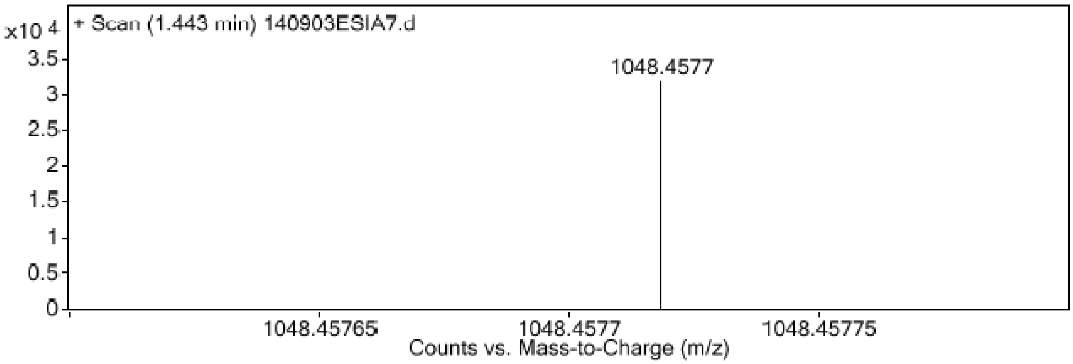

Peak List

m/z	z	Abund
482.1808	1	78736.52

Formula Calculator Element Limits

Element	Min	Max
C	0	200
H	0	400
O	7	15
N	9	9
Na	1	1
S	1	1

Formula Calculator Results

Formula	CalculatedMass	Mz	Diff.(mDa)	Diff. (ppm)	DBE
C52 H67 N9 Na O11 S	1048.4578	1048.4577	0.1	0.1	23.5

--- End Of Report ---

HR-ESI-MS spectrum of **CB12**
